# Individual variability in behavioral flexibility predicts sign-tracking tendency

**DOI:** 10.3389/fnbeh.2015.00289

**Published:** 2015-11-03

**Authors:** Helen M. Nasser, Yu-Wei Chen, Kimberly Fiscella, Donna J. Calu

**Affiliations:** ^1^Behavioral Neuroscience Research Branch, Intramural Research Program, National Institute on Drug Abuse, National Institutes of Health, Department of Health and Human ServicesBaltimore, MD, USA; ^2^Anatomy and Neurobiology, University of Maryland School of MedicineBaltimore, MD, USA

**Keywords:** sign-tracking, outcome devaluation, second-order conditioning, behavioral flexibility, Pavlovian incentive learning

## Abstract

Sign-tracking rats show heightened sensitivity to food- and drug-associated cues, which serve as strong incentives for driving reward seeking. We hypothesized that this enhanced incentive drive is accompanied by an inflexibility when incentive value changes. To examine this we tested rats in Pavlovian outcome devaluation or second-order conditioning prior to the assessment of sign-tracking tendency. To assess behavioral flexibility we trained rats to associate a light with a food outcome. After the food was devalued by pairing with illness, we measured conditioned responding (CR) to the light during an outcome devaluation probe test. The level of CR during outcome devaluation probe test correlated with the rats' subsequent tracking tendency, with sign-tracking rats failing to suppress CR to the light after outcome devaluation. To assess Pavlovian incentive learning, we trained rats on first-order (CS+, CS−) and second-order (SOCS+, SOCS−) discriminations. After second-order conditioning, we measured CR to the second-order cues during a probe test. Second-order conditioning was observed across all rats regardless of tracking tendency. The behavioral inflexibility of sign-trackers has potential relevance for understanding individual variation in vulnerability to drug addiction.

## Introduction

Addiction is a chronically relapsing disorder that develops only in a subset of individuals that engage in recreational drug use. Approximately 15–30% of individuals that try drugs of abuse transition to addiction (Anthony et al., 1994). The behavior of addicted individuals is characterized by a heightened motivation for drugs and an inflexibility characterized by a persistence to seek and take drugs despite negative consequences (American Psychiatric Association, 2013). Animal procedures exploring individual variability in natural reward seeking during Pavlovian lever autoshaping have identified phenotypic behavioral differences that predict vulnerability to cue-driven drug seeking (Tomie, 1996; Flagel et al., 2009; Saunders and Robinson, 2010; Saunders et al., 2013; Yager and Robinson, 2013). Such studies have demonstrated that heightened incentive motivation for natural reward-associated cues serves as an informative predictor of heightened motivation for drug seeking.

During Pavlovian lever autoshaping, where the extension and retraction of a lever precedes the delivery of food reward, rats show individual differences in conditioned responding (CR). Sign-tracking rats preferentially approach and contact the lever, while goal-tracking rats preferentially approach and contact the food cup (Hearst, 1974; Boakes, 1977; Flagel et al., 2007). Sign-tracking rats show heightened cue-directed and/or cue-driven motivation for food-, cocaine-, opioid-, and nicotine- associated cues as compared to goal-tracking rats (Robinson and Flagel, 2009; Flagel et al., 2010; Saunders and Robinson, 2010, 2013; Yager and Robinson, 2010; Saunders et al., 2013; Yager et al., 2015). These findings suggest that sign-trackers show heightened incentive motivation, which is behavior driven by the transfer of reinforcing properties of the reward to the reward-predictive cue, for both natural- and drug-reward associated cues. This heightened incentive motivation driven by drug-associated cues persists in sign-tracking rats even when their drug seeking is punished by an aversive footshock barrier in front of the drug-associated response apparatus (Saunders et al., 2013). This finding suggests that after drug-exposure sign-tracking individuals also fail to appropriately adjust reward seeking in response to punishment. An open question that remains is whether such inflexibility in sign-trackers is driven by too much incentive value attributed to the initially appetitive cue, and/or whether there are deficits in incorporating information about changing incentive value of actions, outcomes, and/or their associated cues.

To begin to address this, in Experiment 1, we examined whether individual variability in behavioral flexibility after outcome devaluation predicts tracking phenotype. Pavlovian outcome devaluation (Holland and Straub, 1979) is a procedure that examines the extent to which a previously reward-paired conditioned stimulus (CS) drives CR after the unconditioned stimulus (US) has been devalued through pairing with illness, and thus is the ideal Pavlovian procedure to examine the flexibility of cue-driven behavior when incentive value of the outcome changes. It is thought that a reduction in CR in the outcome devaluation probe test is driven by stimulus-outcome associations, that is, the ability of the CS to evoke a representation of the current incentive value of the US to drive flexible behavior. However, a failure to display a flexible reduction in CR after outcome devaluation could instead be explained by enhanced incentive value attributed to the initially appetitive cue, for which enhanced stimulus-response associations would effectively mask any stimulus-outcome driven learning after outcome devaluation.

To address this possibility, in Experiment 2, we examined whether individual variability in the expression of previously acquired appetitive incentive value, as assessed by Pavlovian second-order conditioning, predicts the tracking phenotype. Pavlovian second-order conditioning (Pavlov, 1927; Rizley and Rescorla, 1972; Holland and Rescorla, 1975) is a procedure that examines the extent to which a previously reward-paired CS alone is able to support CR to a novel second-order conditioned stimulus (SOCS), and thus is the ideal Pavlovian procedure to examine the expression of previously acquired appetitive incentive value. It is thought that heightened responding to the SOCS is the result of the association between first- and second-order cues (stimulus-stimulus) and/or associations between the second-order cue and conditioned responses previously evoked by the first-order cue (stimulus-response associations), the latter of which is evidence of Pavlovian incentive learning (Rizley and Rescorla, 1972; Rescorla, 1980; McDannald et al., 2013).

After testing rats in Pavlovian outcome devaluation or second-order conditioning procedures we determined their tracking phenotype by screening them in the lever autoshaping procedure. We predicted that a failure to suppress CR after outcome devaluation (Experiment 1) and heightened CR to second-order cues (Experiment 2) would predict the sign-tracking phenotype.

## Materials and methods

### Subjects

Male Long-Evans rats (Charles River Laboratories, Wilmington, MA; 250–325 g at time of arrival; Experiment 1 between subject design; total *n* = 60; Experiment 2 within subject design total *n* = 24) were singly housed and maintained on a reverse 12 h light/dark cycle (lights off at 8 a.m.). Rats had *ad libitum* access to water and standard laboratory chow for 2 days (Experiment 2) and 6 days (Experiment 1) before being food restricted to 85% of their baseline *ad libitum* body weight. Once all rats reached 85% of their baseline body weight they were maintained at 85–90% throughout the behavioral experiments, which were performed in accordance to the “Guide for the care and use of laboratory animals” (8th edition, 2011, US National Research Council). Experimental protocols were approved by the Intramural Research Program (NIDA) Animal Care and Use Committee.

Behavioral experiments were conducted in individual standard experimental chambers (25 × 27 × 30 cm; Med Associates) that were enclosed in a sound-resistant shell. For both experiments, rats were housed in the animal facility, transferred to the experimental chambers prior to the training sessions, and returned to the facility at the end of the sessions.

### Experiment 1: Pavlovian outcome devaluation

#### Apparatus

For Experiment 1, each chamber had one 6 W white cue light located 10 cm above the floor in the center of the wall and a speaker 1 cm above the cue light. A red houselight (6 W bulb covered by red lens) was located at the top of the same wall. The opposite wall was outfitted with a recessed food cup (with photobeam detectors) 2 cm above the floor grid attached to a programed pellet dispenser, which delivered 45 mg food pellets containing 12.7% fat, 66.7% carbohydrate, and 20.6% protein (catalog #1811155; Test Diet 5TUL). The red houselight was illuminated at the start of each training session and was extinguished at the end of each session. Two retractable levers were located on either side of the food cup 6 cm above the floor. These levers remained retracted during the light conditioning phase of the experiment.

#### Phase I: Pavlovian light conditioning

A summary of our experimental design can be found in Table 1. Behavioral training began with a session that reduces the novelty of the unconditioned stimuli. We exposed rats to a single 64 min magazine training session consisting of eight trials in which two 45 mg pellets [Testdiet Purified Rodent Tablet (5TUL)] were delivered (0.5 s apart) on a VI 225 s schedule (200–250 s) for 16 trials. Following magazine training, we trained rats in eight daily 64 min light conditioning sessions, consisting of 16 trials of a 10 s cue light CS. At CS offset two pellets spaced 0.5 s apart were immediately delivered to the food cup on a VI 225 s schedule (200–250 s). We recorded the number of nose pokes and time spent in food cup. We provided chow in the homecage and returned the rats to the animal facility after daily light conditioning sessions.

**Table 1:** **Summary of experimental designs**.

**Experimental group**	**Phase I**	**Phase II**	**Phase III**	**Phase IV**
Experiment 1: Outcome devaluation Between-subjects	Light-food training	Conditioned taste aversion	Test	Lever autoshaping
Paired	A → food	food → LiCl	A	L1 → suc; L2
Unpaired	A → food	food; LiCl	A	L1 → suc; L2
Experiment 2: Second-order conditioning Within-subjects	Light-food training	Second-order training	Test	Lever autoshaping
	A → food; B	X → A; Y → B; A → food; B	X; Y; A; B	L1 → suc; L2

*The right arrow (→) signifies paired presentations. A and B are visual conditioned stimuli (for Experiment 1 we used a steady light and for Experiment 2 we used steady or blinking cue lights, counterbalanced for CS+ and CS−), X and Y were auditory stimuli (white noise or a tone, counterbalanced for SOCS+ and SOCS−), L1 refers to lever 1 and L2 referred to lever 2 (location counterbalanced), food signifies the delivery of two food pellet US; LiCl signifies lithium chloride injections, suc signifies the delivery of 0.5 mL of 10% sucrose*.

#### Phase II: conditioned taste aversion training

One day after the final light conditioning session, we devalued the pellets used during light conditioning in a homecage conditioned taste aversion (CTA) procedure that took place in rats' homecage over 4 days. We divided the rats into paired (*n* = 30) and unpaired (*n* = 30) groups. We habituated all rats in their homecage to the ceramic ramekins used to present food pellets during subsequent CTA procedures. Across the 4 days of CTA, we exposed all rats to both pellets and lithium chloride (LiCl)-induced gastric malaise in order to equate experience between the paired and unpaired groups; however explicit pairing of pellets with illness occurred only in the paired group. On the first and third days of CTA, we gave paired rats 10 min of homecage access to 100 pellets in ceramic ramekins followed immediately by LiCl injection (0.3 M, 5 mL/kg, i.p.), whereas we gave the unpaired rats only the LiCl injection (0.3 M, 5 mL/kg, i.p.). On the second and fourth days, we gave unpaired rats 10 min of homecage access to 100 pellets in ceramic ramekins, and paired rats remained in the homecage with no intervention. We gave all rats standard homecage chow (amount based on 85% body weight with compensation for CTA pellet consumption) 6 h after pellet access and/or injections each day during this phase of the experiment to prevent association of LiCl-induced illness with homecage chow.

#### Phase III: outcome devaluation probe test

One day after the final day of the CTA procedure, we conducted an outcome devaluation probe test. During this 64 min extinction session, the 10 s cue light was illuminated on a VI 90 s schedule (60–120 s) for 16 trials, but no pellets were delivered. We recorded time spent in the food cup during the pre-CS (10 s in 5 s bins), CS (10 s in 5 s bins), post-CS period (1.5 s post-CS when reward was previously delivered), and post-reward (5 s post reward). Three hours after the probe test, we gave rats 10 min access to 50, 45 mg pellets (same as the US used during conditioning and in CTA), which we placed in the magazine of operant chambers and we recorded the number of pellets consumed to assess generalization of taste aversion from homecage to experimental chamber. We performed a post-probe test homecage consumption test the next day, in which we gave all rats 10 min homecage access to 100 pellets, and we recorded the number of pellets consumed. Rats that had pellet consumption that fell three standard deviations outside of the group mean during homecage or chamber tests were excluded from the study (paired *n* = 2, unpaired *n* = 1).

#### Phase IV: lever autoshaping procedure (sign-tracking screening procedure)

After the outcome devaluation probe test, in order to reduce the novelty of the sucrose US, we gave rats a single 75 min magazine training session, during which 0.5 mL of 10% sucrose was delivered into the food cup on a VI 90 s schedule (60–120 s) for 25 trials. Subsequently, we trained rats for 5 days in autoshaping sessions (~75 min per session) in which there were 25 CS+ and 25 CS− presentations occurring on a VI 90 s schedule (60–120 s). CS+ trials consisted of the insertion of a retractable lever (left or right, counterbalanced) paired with a 5150 Hz tone for 10 s, after which the lever was retracted and 0.5 mL of sucrose was delivered to the food cup. CS− trials consisted of the insertion of a retractable lever (left or right, counterbalanced) paired with a 12,163 Hz tone for 10 s, after which the lever was retracted and no reward delivered.

### Experiment 2: Pavlovian second-order conditioning

#### Apparatus

For Experiment 2, each experimental chamber had two 6 W white cue lights located 10 cm above the floor to the left and right of a recessed food cup (with photobeam detectors) 2 cm above the grid floor on the center of the wall attached to a programed pellet dispenser, which delivered 45 mg food pellets containing the same pellet as used in Experiment 1 (catalog #1811155; Test Diet 5TUL). In the center of the opposite wall a speaker was located 8 cm above the floor. A red houselight (6 W bulb covered by red lens) was located at the top of same wall. The red houselight was illuminated at the start of each training session and was extinguished at the end of each session.

#### Phase I: Pavlovian light conditioning (first-order conditioning)

A summary of our experimental design can be found in Table 1. Behavioral training began with two sessions that reduce the novelty of the unconditioned and conditioned stimuli. We exposed rats to a single 64 min magazine training session consisting of eight trials in which two 45 mg pellets (Testdiet Purified Rodent Tablet 5TUL) were delivered 0.5 s apart to the food cup on a VI 240 s schedule (60–420 s intervals). The same day we exposed rats to a single 32 min light pre-exposure session consisting of four presentations of each of the two cue lights (flashing and steady) and no pellets on a VI 240 s schedule (60–420 s intervals). After magazine training and light pre-exposure sessions, we gave rats 12 daily 64 min light conditioning sessions, consisting of 16 trials. Each session consisted of eight CS+ trials, in which a 10 s cue light (flashing or steady light) was rewarded with two pellets delivered on the 9th and 10th second of the CS (stimulus type was counterbalanced across rats) and eight CS− trials, in which the alternate cue light (flashing or steady light) was not rewarded. These lights were counterbalanced by side (left or right of the food cup) and stimulus (flashing or steady). Presentation of CS+ and CS− trials were intermixed to enhance discrimination between the two cue lights. We recorded the number of food cup entries during the conditioning phase. We provided chow in the homecage and returned the rats to the animal facility after daily light conditioning sessions.

#### Phase II: second-order conditioning

After 12 days of first-order conditioning (FOC), to reduce the novelty of the second-order conditioned stimuli, we exposed rats in a single 32 min pre-exposure session consisting of eight intermixed trials of second-order cues: four trials of a 10 s tone (5150 Hz, 2 dB) cue presentation, and four trials of 10 s white noise (82 dB) cue presentation. After the pre-exposure session, second-order conditioning started. Second-order conditioning involved three 64 min daily sessions of 16 trials on a VI 240 s schedule (60–420 s intervals). During second-order conditioning there were two types of FOC cues presented within a session. Elemental FOC trials served as “reminder” trials of Pavlovian first-order light discrimination conditioning, and were simply reward-paired first order CS+ trials and unrewarded CS− trials that were presented to remind rats of the discrimination between first-order stimuli during the second-order conditioning phase. Compound FOC trials were simply the second-order trials in which auditory SOCS+ or SOCS− cues paired with respective first-order CS+ or CS−. There were eight first-order cue “elemental” reminder trials and there were eight second-order conditioning trials: four trials of low tone immediately followed by a 10 s light cue (flash or steady) and four trials of noise immediately followed by the alternate 10 s light cue (flash or steady) with order of stimuli counterbalanced. During second-order conditioning sessions we recorded the number of food cup entries.

#### Phase III: second-order conditioning probe test

After 3 days of second-order conditioning, we exposed rats to a single 64 min probe test session consisting of 16 trials presented on a VI 240 s schedule (60–420 s intervals). There were eight intermixed first-order cue presentations (CS+ and CS− light cues) and separately eight intermixed second-order cue presentations (SOCS+ and SOCS− auditory cues) all presented alone, with the second-order cues presented in a block prior to first-order cues. During second-order conditioning probe test, we recorded the number of food cup entries and video for later scoring of rearing and head jerk behaviors as described below in the response measures section.

#### Phase IV: lever autoshaping procedure (sign-tracking screening procedure)

After the second-order conditioning probe test, in order to reduce the novelty of the sucrose US, we exposed rats to a single 75 min magazine training session, during which 0.5 mL of 10% sucrose was delivered into the food cup on a VI 90 s schedule (60–120 s) for 25 trials. Subsequently, we gave rats five sessions of autoshaping (~75 min per session) in which there were 25 CS+ and 25 CS− presentations occurring on a VI 90 s schedule (60–120 s). CS+ trials consisted of the insertion of a retractable lever (left or right, counterbalanced) for 10 s, after which the lever was retracted and 0.5 mL of sucrose was delivered to the food cup. CS− trials consisted of the insertion of a retractable lever (left or right, counterbalanced) paired for 10 s, after which the lever was retracted and no reward delivered. At the start of each session the red houselight is turned on and at the end of each session the houselight is turned off.

#### Response measures

##### Food cup behavior

For Experiment 1, Pavlovian outcome devaluation Phases I and III, the primary measure of appetitive conditioning was the percentage of time the rat spent in the food cup during the 10 s CS and during the 10 s interval immediately before each CS (pre-CS), as determined by interruption of the photocell beam in the magazine. For the outcome devaluation probe test we also measured the percentage of time the rat spent in the food cup during the 1.5 s post-CS (time when reward was delivered during conditioning). For Experiment 2, Pavlovian second-order conditioning Phases I-III, the primary measure of appetitive conditioning was the number of food cup responses during the 10 s CS and during the 10 s interval immediately before each CS (pre-CS), as determined by interruption of the photocell beam in the magazine. Previous studies (Holland, 1977) have indicated that in FOC most food cup behavior occurs during the last 5 s of a 10 s CS, and thus we examined data in 5 s bins.

##### Pellet consumption

For Experiment 1, Outcome devaluation, the consumption of food pellets during taste aversion training and subsequent devaluation tests was determined by counting the number of pellets remaining in the ramekin and bedding after 10 min (for homecage test) or by counting the number of pellets remaining in the magazine and tray beneath the experimental chamber floor after 10 min (in chamber test).

##### Rearing and head jerk scored from video

Two experimenters were blinded to the experimental groups and independently scored a subset (33%) of the same videos to confirm accuracy and consistency of video scoring for rearing and head jerk measures. Video scores obtained by each individual were significantly correlated for both measures (rearing *r*^2^ = 0.86, *p* < 0.001; head jerk *r*^2^ = 0.91, *p* < 0.001).

##### Rearing behavior

For Experiment 2, Second-order conditioning Phase III, rearing (conditioned orienting observed to light stimuli) was a second measure of appetitive conditioning. We observed and scored rearing behavior in one-second intervals for the pre-cue (10 s) and cue (10 s) periods. Rearing was defined as standing with both front forepaws off of the grid (Holland, 1977).

##### Head jerk behavior

For Experiment 2, Second-order conditioning Phases III, head jerk (conditioned orienting observed to auditory stimuli) was a third measure of appetitive conditioning to first- and second-order cues. We observed and scored head jerk behavior in 1 s intervals for the pre-cue (10 s) and cue (10 s) periods. Head jerk was defined as short rapid horizontal or vertical movements of the head usually oriented toward the food magazine or source of the audio output. Simultaneous display of head jerk and hindquarter movement or rearing, were scored as head jerk or not head jerk (Holland, 1977).

##### Lever autoshaping behavioral measures

Behavioral characterization of sign- and non-sign trackers were identical for Experiments 1 and 2 and were based on a Pavlovian Conditioned Approach analysis (Meyer et al., 2012). The primary measure of tracking tendency was characterized by the average of three difference score measures that make up the composite sign-tracking score that ranges from −1.0 to +1.0. These three difference score measures of sign-tracking behavior are: (1) preference score, (2) latency score, and (3) probability score, which were calculated for each lever autoshaping session. The preference score (ranges from −1.0 to 1.0) was the number of lever presses during the CS, minus the number of food cup responses during the CS, divided by the sum of these two measures. The latency score (ranges from −1.0 to 1.0) was the average latency to make a food cup response during the CS, minus the latency to lever press during the CS, divided by the duration of the CS (10 s). The probability score (ranges from −1.0 to 1.0) was the probability the rat will lever press minus the probability the rat will make a food cup response, determined on a trial by trial basis and averaged across the session to determine probability of each response. The composite ST score (ranges from −1.0 to 1.0) was determined for each session and was the average of the preference score, latency score, and probability score. Sign-tracking (ST) was defined by a composite score ranging from +0.5 to +1.0 and non-sign tracking (non-ST) was defined by a score ranging from +0.49 to −1.0, and was comprised of intermediate rats with scores ranging from +0.49 to −0.49 and goal-tracking rats with scores ranging from −0.5 to −1.0. Generally speaking, sign-tracking rats prefer and press the lever at a higher frequency, shorter latency, and higher probability than they respond at the food cup. Goal-tracking rats prefer and respond at the food cup more frequently, at a shorter latency, and higher probability than they respond at the lever. Intermediate rats tend not to have a clear preference for the lever or the food cup, responding at similar levels, latencies and probabilities at the food cup and lever. The final composite tracking score used to characterize the individual rats as ST (≥0.5) or non-ST (< 0.5) was the average composite tracking score across sessions 3–5 of autoshaping.

#### Statistical analyses

The behavioral data were analyzed using the SPSS statistical software (IBM) by ANOVAs and *t*-tests, and significant main effects and interaction effects (*p* < 0.05) were followed by Bonferroni *post-hoc* tests. All statistical analyses of the food cup behavior were done on the raw data counts or number of entries. The dependent measures and the factors used in the statistical analyses are described in the results section below.

## Results

### Experiment 1: Pavlovian outcome devaluation

#### Phase I: Pavlovian light-food conditioning

**Figure 1:**
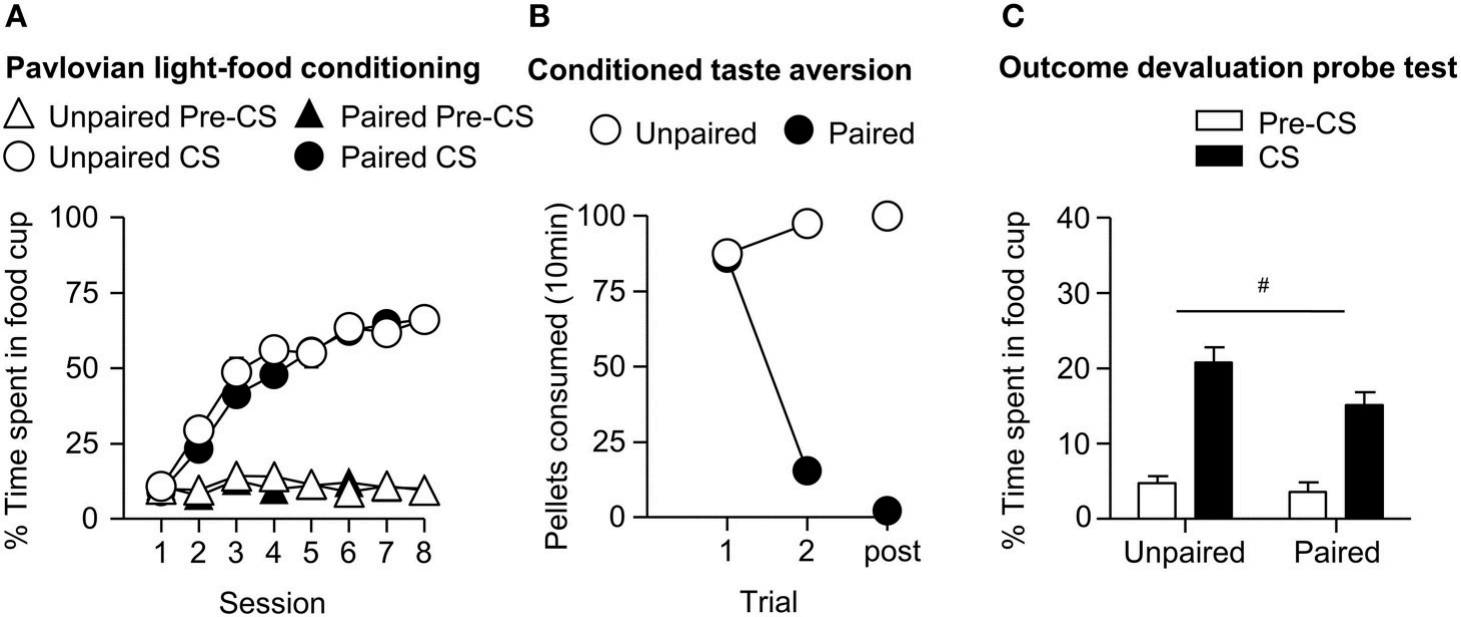
**Experiment 1: Phase I–III: Performance during Pavlovian light-food conditioning, conditioned taste aversion and outcome devaluation probe test**. **(A)** Phase I: Light-food conditioning separated by later determined between subjects factor of Pairing: Paired (*n* = 28) vs. Unpaired (*n* = 29). Percent time spent in food cup (mean ± SEM) during the last 5 s of the 10 s Pre-CS and CS period of light-food conditioning. **(B)** Phase II: Pellet consumption during conditioned taste aversion training and post-probe homecage consumption test. Number of pellets consumed (mean ± SEM) in 10 min conditioned taste aversion training sessions (trial 1 and 2) and during post-probe homecage consumption test session (post). **(C)** Phase III: Overall effect of outcome devaluation during probe test. Percent time spent in food cup (mean ± SEM) during the 10 s light CS and Pre-CS period. Paired (*n* = 28) vs. Unpaired (*n* = 29). ^#^Different in % time spent in food cup between Paired and Unpaired groups, *p* = 0.06.

We trained all rats that a light predicted delivery of a food reward. All rats increased their food cup entries in response to the light CS over the course of eight training sessions, while the response during the pre-CS period remained low and relatively stable. This response curve did not differ between the later determined paired and unpaired groups (Figure 1A). We analyzed the data using a mixed ANOVA, with between subject factor of Pairing (Unpaired, Paired) and within subject factors of Session (1-8), and CS epoch (pre-CS last 5 s, CS last 5 s). There was a main effect of Session [*F*_(7, 385)_ = 80.8, *p* < 0.05] and CS epoch [*F*_(1, 55)_ = 453.8, *p* < 0.05], as well as Session × CS epoch interaction [*F*_(7, 385)_ = 132.8, *p* < 0.05], but no main effect of Pairing [*F*_(1, 55)_ = 0.6, *p* = 0.43] nor interaction of Pairing × Session [*F*_(7, 385)_ = 1.7, *p* > 0.05], Pairing × CS epoch [*F*_(1, 55)_ = 0.4, *p* > 0.05] or Pairing × Session × CS epoch [*F*_(7, 385)_ = 0.9, *p* > 0.05].

#### Phase II: conditioned taste aversion training

After light-food conditioning, homecage exposure to the food and illness were either Paired or Unpaired for the two groups. Paired rats readily reduced consumption of the pellets as compared to unpaired rats (Figure 1B). We analyzed data using a mixed ANOVA with between subjects factor of Paring (Unpaired, Paired) and within subjects of Trial (Day 1, Day 2), in which we found a main effect of Pairing [*F*_(1, 55)_ = 167.9, *p* < 0.05] and Trial [*F*_(1, 55)_ = 211.7, *p* < 0.05] and a Pairing × Trial interaction [*F*_(1, 55)_ = 373.5, *p* < 0.05].

#### Phase III: outcome devaluation probe test

After taste aversion training, we assessed conditioned food cup responding to the light under extinction conditions (Figure 1C). In accordance with other devaluation studies (Pickens et al., 2003), we presented only results from the first eight probe test trials. Data for CS and pre-CS responding were analyzed using mixed ANOVAs with between subject factor of Pairing (Unpaired, Paired) and within subject factor of CS epoch (pre-CS, CS). The analysis of the 10 s pre-CS and CS periods showed that there was a main effect of CS [*F*_(1, 55)_ = 155.8, *p* < 0.05] and a nearly significant main effect of Pairing [*F*_(1, 55)_ = 3.7, *p* = 0.06]. There was no significant interaction of CS epoch and Pairing [*F*_(1, 55)_ = 2.57, *p* = 0.12]. Overall, we found a modest reduction in food cup behavior during the light cue in the Paired group, consistent with the reduced value of the food outcome established during the CTA phase.

To further confirm the strength of aversion to the pellets after the critical probe test we analyzed data from two additional consumption tests in the absence of LiCl injections; one in the homecage (Figure 1B post) and the other in the experimental chamber (data not shown). We analyzed the consumption data from the post-probe homecage test using an ANOVA, with between subject factor of Pairing (Unpaired, Paired). There was a main effect of Pairing [*F*_(1, 55)_ = 8125.0, *p* < 0.05]. We analyzed the consumption data from the post-probe chamber test using an ANOVA, with between subject factors of Pairing (Unpaired, Paired). There was a main effect of Pairing [*F*_(1, 55)_ = 4434.4, *p* < 0.05; data not shown; mean ± SEM; Unpaired = 50.0 ± 0.03, Paired = 2.3 ± 0.7].

#### Phase IV: lever autoshaping procedure (sign-tracking screening procedure)

**Figure 2:**
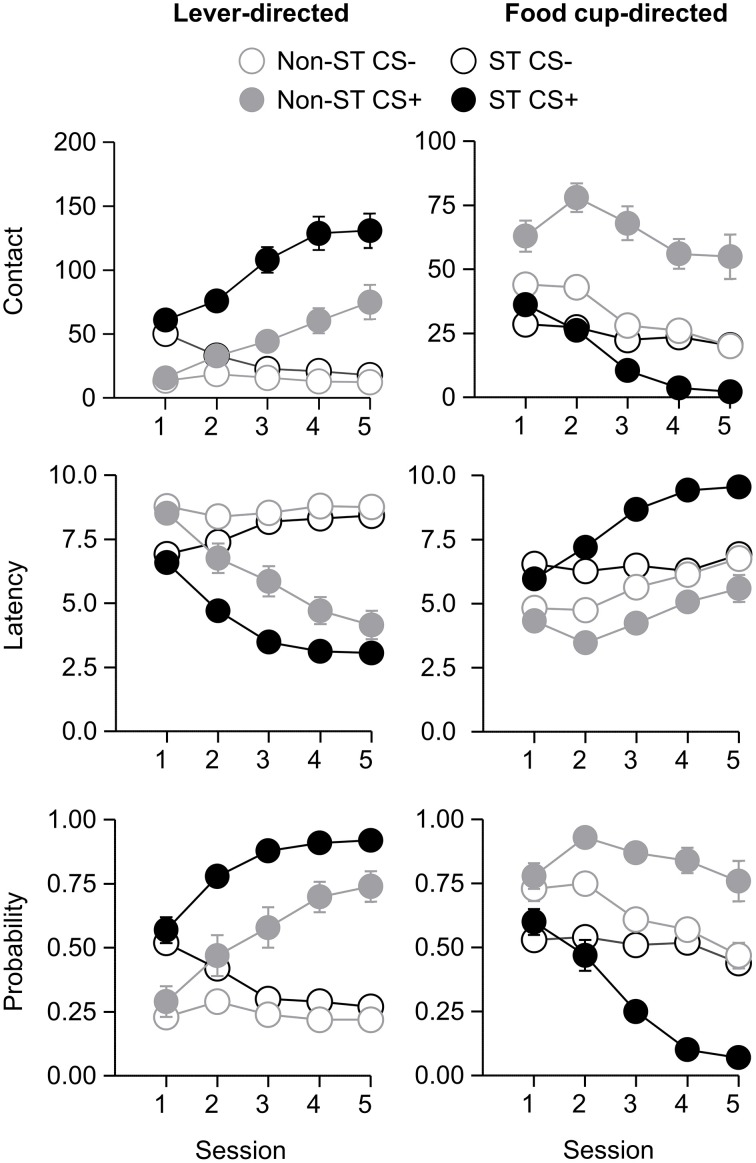
**Experiment 1: Phase IV: Performance during lever autoshaping procedure (Sign-tracking screening procedure)**. Data are mean ± SEM on three different lever-directed (left) and food cup-directed (right) measures. Number of lever and food cup contacts (top row), latency to contact lever or food cup (middle row) and probability of contacting lever or food cup (bottom row).

After the probe test, we screened rats in the lever autoshaping procedure to determine their Tracking tendency. Rats' performance on three lever measures (contact, latency, and probability) and three food cup measures (contact, latency, and probability) is shown in Figure 2. We analyzed the data using six separate sets of mixed repeated measures ANOVAs, using and between subjects factor of Tracking group (non-ST, ST) and within subject factors of CS (CS−, CS+) and Session (1-5). The six analyses were on three lever measures (contact, latency, and probability) and three food cup measures (contact, latency, and probability). The main effects and interactions are reported in Table 2. Importantly, the critical CS × Session × Tracking group interactions were significant for all six measures of CR.

**Table 2:** **Experiment 1. Phase IV: Lever autoshaping procedure, summary table of analyses for lever and food cup measures (contact, latency, and probability)**.

**Effect**	**Degrees of freedom**	**Lever**						**Food cup**					
		**Contact**		**Latency**		**Probability**		**Contact**		**Latency**		**Probability**	
		***F***	***p***	***F***	***p***	***F***	***p***	***F***	***p***	***F***	***p***	***F***	***p***
CS	(1,55)	74.70	< 0.001	271.71	< 0.001	322.80	< 0.001	27.30	< 0.001	3.07	0.09	0.00	0.988
Session	(4, 220)	10.45	< 0.001	22.81	< 0.001	9.30	< 0.001	25.30	< 0.001	24.88	< 0.001	22.76	< 0.001
Tracking group	(1, 55)	23.35	< 0.001	17.91	< 0.001	29.33	< 0.001	102.39	< 0.001	80.29	< 0.001	96.62	< 0.001
CS × Session	(4, 220)	43.70	< 0.001	94.09	< 0.001	83.40	< 0.001	3.19	0.014	9.07	< 0.001	4.13	0.003
CS × Session × Tracking	(4, 220)	4.60	0.001	3.12	0.016	3.53	0.008	12.57	< 0.001	16.78	< 0.001	25.53	< 0.001

### Individual differences in outcome devaluation probe test

**Figure 3:**
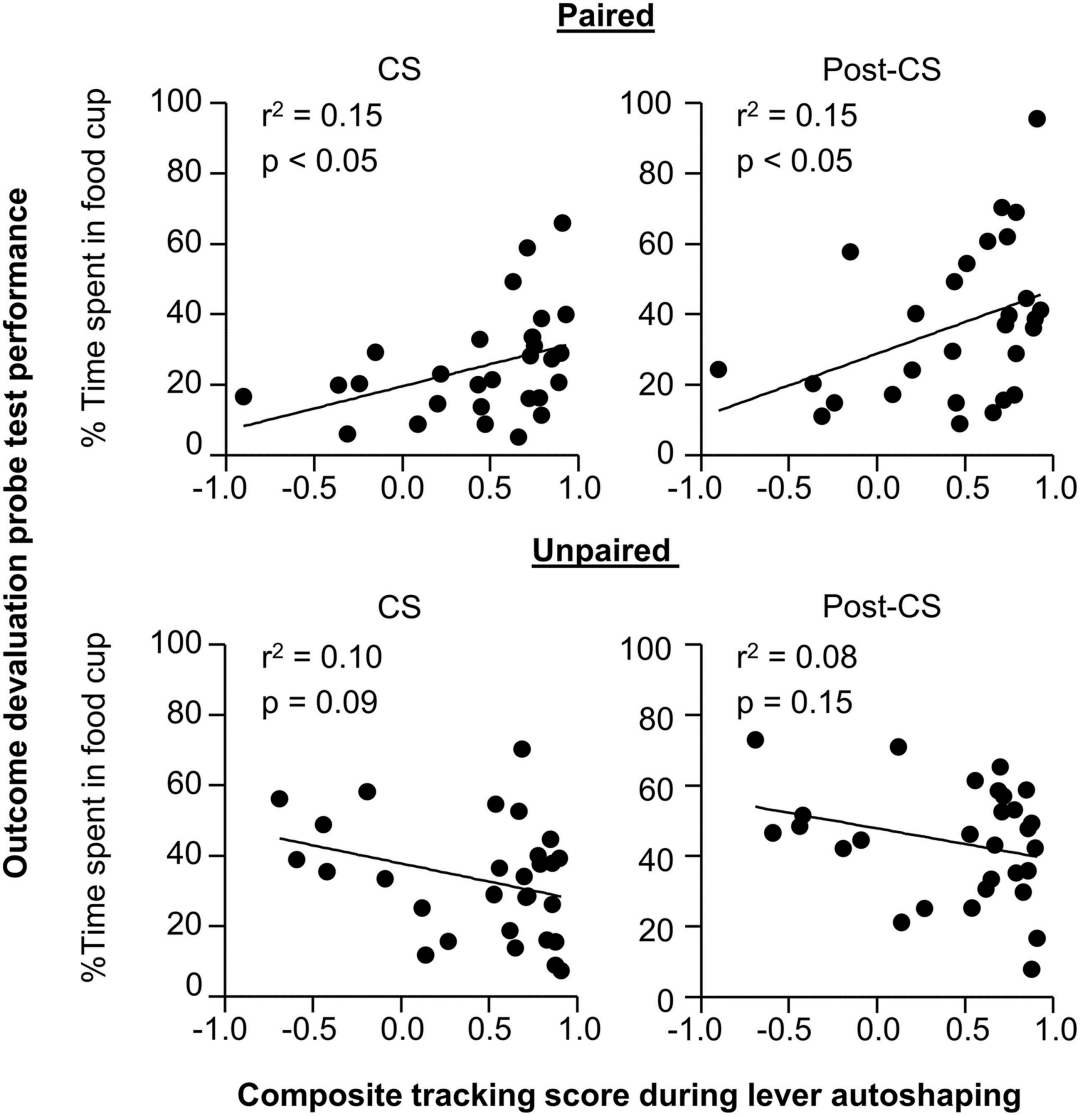
**Experiment 1: Phase IV: Correlation between performance during outcome devaluation probe test with tracking tendency during lever autoshaping**. Correlation for Paired (top row) and Unpaired (bottom row) groups between percent time spent in food cup during outcome devaluation probe test (last 5 s of the CS) with composite tracking score determined from lever autoshaping (left) and between percent time spent in food cup during outcome devaluation probe test post-CS period with composite tracking score determined from lever autoshaping (right).

In order to understand whether performance of individual rats in outcome devaluation relates to tracking tendency we conducted linear correlation analyses using performance during the outcome devaluation probe test and the composite tracking score (Figure 3). For paired rats we observed a significant positive correlation between food cup CR in the last 5 s of the CS during outcome devaluation probe test and the later determined tracking score (*r*^2^ = 0.15, *p* < 0.05; Figure 3 top right), paired rats that responded at highest levels to the devalued stimulus tended to fall toward the sign-tracking end of the continuum. The relationship between food cup CR during the Post-CS period of outcome devaluation probe test and the tracking score was also positively correlated (*r*^2^ = 0.15, *p* < 0.05; Figure 3 top right). There was no such relationship between food cup CR during the pre-CS of outcome devaluation probe test and the tracking score (*r*^2^ = 0.02, *p* = 0.3; data not shown). We also did not observe any correlations in the Unpaired group during the CS or Post-CS period (*r*^2^ = 0.10, *p* = 0.09 and *r*^2^ = 0.08, *p* = 0.15, respectively; Figure 3 bottom). These results suggest that rats that respond more to the CS after outcome devaluation go on to engage in more sign-tracking behaviors during Pavlovian lever autoshaping.

Based on this behavioral correlation, we conducted further statistical analyses of each phase of Experiment 1 now including the between subject factor of Tracking tendency to confirm the predictive relationship between flexibility in response to devalued outcomes and tracking tendency. To account for the possibility of unequal variance between our tracking groups for the CS and reward period during probe test we ran the Levene's test for equality of variance [*F*-critical_(1, 55)_ = 5.3, type I error rate α = 0.05]. Food cup responding for paired non-sign-tracking and sign-tracking rats was less than the F-critical [*F*_(1, 55)_ = 3.9, *p* = 0.06 and *F*_(1, 55)_ = 1.5, *p* = 0.23, CS and Post-CS period, respectively]. Therefore, there was insufficient evidence for unequal variance between non-sign-tracking and sign-tracking paired rats in food cup responding, so degrees of freedom did not need adjustment for the following analyses.

**Figure 4:**
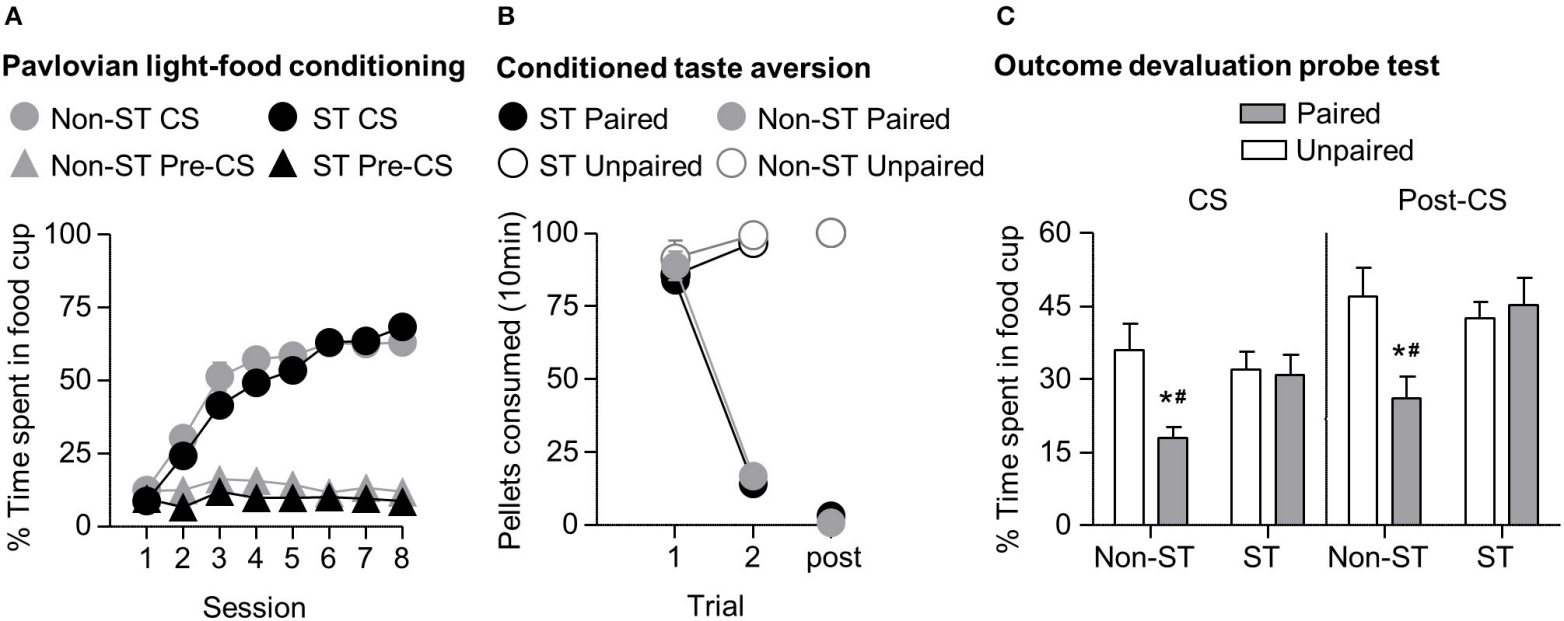
**Experiment 1: Phase I–III: Performance during Pavlovian light-food conditioning, conditioned taste aversion and outcome devaluation probe test separated by tracking tendency**. **(A)** Phase I: Light-food conditioning separated by between subjects factor Tracking tendency: sign-tracking (*n* = 36) vs. non-sign-tracking (*n* = 21). Percent time spent in food cup (mean ± SEM) during the last 5 s of the 10 s Pre-CS and CS period of light-food conditioning. **(B)** Phase II: Pellet consumption during conditioned taste aversion training and post-probe homecage consumption test. Number of pellets consumed (mean ± SEM) in 10 min conditioned taste aversion training sessions (trial 1 and 2) and during post-probe homecage consumption test session (post). Paired or Unpaired data is separated by the later determined between subjects factor of tracking tendency. **(C)** Phase III: Overall effect of outcome devaluation during probe test. Percent time spent in food cup (mean ± SEM) during outcome devaluation probe test separated by tracking tendency during CS (left) and post-CS (right) for Unpaired and Paired groups. ^*^Different percent time spent in food cup between Unpaired and Paired groups within tracking tendency, *p* < 0.05. Paired non-sign-tracking (*n* = 12); Paired sign-tracking (*n* = 16) vs. Unpaired non-sign-tracking (*n* = 9); Unpaired sign-tracking (*n* = 20). ^#^Different in % time spent in food cup between Paired sign-trackers and Paired non-sign-trackers groups, *p* = 0.05.

In phase I of light-food training, all rats increased their food cup entries in response to the light CS over the course of eight training sessions, and this response curve did not differ between the later determined sign-tracking and non-sign-tracking tendency groups (Figure 4A) We analyzed the data using a mixed ANOVA, with between subject factor of Tracking tendency (non-ST, ST) and within subject factors of Session (1-8), and CS epoch (pre-CS last 5 s, CS last 5 s). Three was a main effect of Session [*F*_(7, 385)_ = 72.8, *p* < 0.05] and CS epoch [*F*_(1, 55)_ = 418.3, *p* < 0.05], as well as Session × CS epoch interaction [*F*_(7, 385)_ = 120.9, *p* < 0.05] but no main effect of Tracking tendency [*F*_(1, 55)_ = 3.3, *p* = 0.08], and no interactions of Tracking tendency × Session [*F*_(7, 385)_ = 1.7, *p* > 0.05], Tracking tendency × CS epoch [*F*_(1, 55)_ < 0.1, *p* > 0.05], or Tracking tendency × Session × CS epoch [*F*_(7, 385)_ = 1.5, *p* > 0.05].

In phase II, the CTA developed similarly in both sign-tracking and non-sign-tracking rats (Figure 4B). For taste aversion training we analyzed the data using a mixed ANOVA, between subjects factors of Pairing (Unpaired, Paired) and Tracking tendency (non-ST, ST) and within subject factor of Trial (Day 1, Day 2). We found main effects of Pairing [*F*_(1, 53)_ = 155.9, *p* < 0.05] and Trial [*F*_(1, 53)_ = 192.5, *p* < 0.05] and a significant interaction of Trial × Pairing [*F*_(1, 53)_ = 328.2, *p* < 0.05]. There were no significant main effects of Tracking tendency [*F*_(1, 53)_ = 1.43, *p* = 0.24]. Nor were there interactions of Tracking tendency with Pairing [*F*_(1, 53)_ < 0.01, *p* = 0.96] Trial [*F*_(1, 53)_ = 0.36, *p* = 0.55], or Trial × Pairing [*F*_(1, 53)_ < 0.01, *p* = 0.94].

In phase III, we focused our analysis to food cup behavior during the last 5 s of the CS. We found that the non-sign-tracking paired rats reduced their food cup responding, while paired and unpaired sign-trackers showed similar levels of food cup responding in probe test (Figure 4C). We analyzed this using a mixed ANOVA with between subject factors of Pairing (Unpaired, Paired) and Tracking tendency (non-ST, ST) and a within subject factor of CS epoch (pre-CS, CS). We found main effects of CS [*F*_(1, 53)_ = 140.5, *p* < 0.05] and Pairing [*F*_(1, 53)_ = 6.2, *p* < 0.05], as well as a Pairing × Tracking tendency interaction [*F*_(1, 53)_ = 6.2, *p* < 0.05]. One-way ANOVA of food cup behavior during the Post-CS period, during which time the food pellets were previously delivered in the conditioning phase, showed the same critical Pairing × Tracking tendency interaction [*F*_(1, 53)_ = 6.2, *p* < 0.05]. Bonferroni *post-hoc* comparisons confirmed that paired ST rats spent more time in the food cup relative to paired non-ST rats during the CS and Post-CS periods [*F*_(1, 53)_ = 5.03, *p* < 0.05 and *F*_(1, 53)_ = 7.71, *p* < 0.05, respectively]. That is, sign-tracking paired rats were less flexible than non-sign-tracking paired rats.

Notably, we did not see evidence for extinction learning differences between tracking groups when repeating the above analysis on food cup responding during the CS with the additional factor of Time (Trial 1–4, Trial 5–8) (data not shown). Importantly, the difference between non-ST paired and unpaired groups was evident in the first trial block (Time × Pairing × Sign-tracking tendency [*F*_(1, 53)_ = 4.7, *p* < 0.05; mean ± SEM non-ST Unpaired: 51.3 ± 9.4%; non-ST Paired: 26.2 ± 3.1%, *post-hoc*: *p* < 0.05]), which was not true for ST paired and unpaired (ST Unpaired: 42.7 ± 4.2%; ST Paired: 46.0 ± 5.6%, *post-hoc*: *p* > 0.05). In addition, both tracking groups extinguished at a similar rate, as evidenced by failure to see interaction for Time × Tracking [*F*_(1, 53)_ = 0.186, *p* > 0.05] and by similar terminal levels of food cup responding between both unpaired groups in the second block of trials (mean ± SEM non-ST Unpaired: 20.6 ± 3.0%; ST Unpaired: 21.3 ± 4.0%; non-ST Paired: 9.5 ± 3.0%; ST Paired: 15.70 ± 3.7%). Overall, this suggests that the sign-trackers display less flexible behavior after reward devaluation than non-sign-trackers.

To further confirm the strength of aversion to the pellets after the critical probe test we performed two additional consumption tests in the absence of LiCl injections; one in the homecage (Figure 1C, post) and the other in the experimental chamber. We analyzed the consumption data from the post-probe homecage test using an ANOVA, with between subject factors of Pairing (Unpaired, Paired) and Tracking tendency (non-ST, ST). There was a main effect of Pairing [*F*_(1, 53)_ = 7478.0, *p* < 0.05], but no main effect of Tracking tendency [*F*_(1, 53)_ = 1.0, *p* > 0.05] and no interaction effect of Pairing and Tracking [*F*_(1, 53)_ = 1.0, *p* > 0.05]. We analyzed the consumption data from the post-probe chamber test using an ANOVA, with between subject factors of Pairing (Unpaired, Paired) and Tracking tendency (non-ST, ST). There was a main effect of Pairing [*F*_(1, 53)_=4105.3, *p* < 0.05; data not shown; mean ± SEM; Unpaired non-ST = 50 ± 0.0, Unpaired ST = 50 ± 0.1, Paired non-ST = 1.4 ± 0.7, Paired ST = 3.0 ± 1.1], but no significant main effect of Tracking tendency [*F*_(1, 53)_ = 1.1, *p* > 0.05] or interaction of Pairing and Tracking [*F*_(1, 53)_ = 1.2, *p* > 0.05].

### Experiment 2: Pavlovian second-order conditioning

#### Phase I: Pavlovian first-order light discrimination conditioning

**Figure 5:**
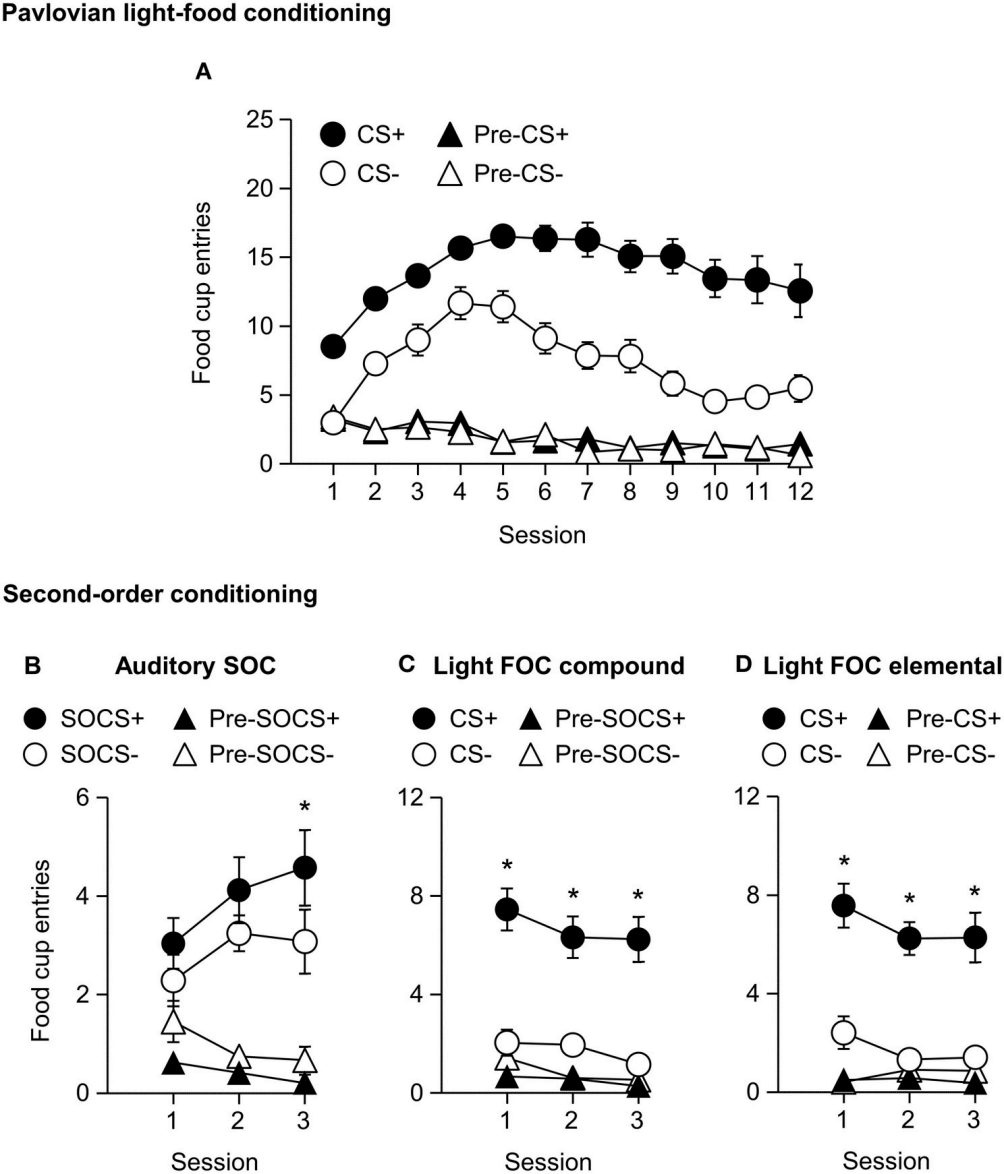
**Experiment 2: Phase I–II: Performance during Pavlovian first-order light discrimination conditioning and second-order conditioning**. **(A)** Phase I: Performance during Pavlovian first-order light discrimination conditioning. Number of food cup entries (mean ± SEM) during the last 5 s of the 10 s Pre-CS and CS periods of a rewarded light CS+ (blinking or steady, counterbalanced) predictive of food or an unrewarded light CS− (blinking or steady, counterbalanced) predictive of no food. **(B–D)** Experiment 2: Phase II: Performance during Pavlovian second-order auditory discrimination conditioning. **(B)** Number of food cup entries (mean ± SEM) during the total 10 s of the Pre-SOCS and SOCS periods for the auditory second-order cues (SOCS+ or SOCS−). **(C)** Number of food cup entries (mean ± SEM) during the last 5 s of the 10 s Pre-CS and CS for the light first-order cues (FOC compound; CS+ or CS−) when it was presented in compound with the SOCS+ or SOCS−. **(D)** Number of food cup entries (mean ± SEM) during the last 5 s of the 10 s Pre-CS and CS for the light first-order cues (FOC element; CS+ or CS−) element, “reminder trials,” when these light cues were rewarded or unrewarded, respectively. ^*^Different in mean number of food cup entries between SOCS+ vs. SOCS− or CS+ vs. CS−, *p* < 0.05.

We trained all rats in a first-order light CS+, CS− discrimination. All rats increased food cup entries in response to the CS+ over the course of 12 training sessions. In comparison food cup entries in response to the CS− over the course of 12 training sessions were lower than during the CS+ and remained relatively stable. Conditioned food cup entries during both the pre-CS+ and pre-CS− periods were low to start and remained relatively stable (Figure 5A). We focused our analysis on food cup entries during the last 5 s of the CS, a time when most food cup behavior occurs during FOC (Holland, 1977, 1980). We analyzed the data using a within subjects repeated measures ANOVA, using factors of Session (1–12), CS epoch (Pre-CS, CS), and CS Discrimination (CS−, CS+). There were main effects of Session [*F*_(11, 253)_ = 8.7, *p* < 0.05], CS epoch [*F*_(1, 23)_ = 353.2, *p* < 0.05], and CS Discrimination [*F*_(1, 23)_ = 132.3, *p* < 0.05]. There were significant interactions of Session × CS epoch [*F*_(11, 253)_ = 22.8, *p* < 0.05], Session × CS Discrimination [*F*_(11, 253)_ = 12.0, *p* < 0.05] and CS epoch × CS Discrimination [*F*_(1, 23)_ = 200.4, *p* < 0.05] as well as a significant Session × CS epoch × CS Discrimination interaction [*F*_(11, 253)_ = 11.8, *p* < 0.05].

#### Phase II: Pavlovian second-order auditory discrimination conditioning

After first-order light discrimination conditioning, we trained all rats in a second-order auditory SOCS+, SOCS− discrimination (Figures 5B–D). We focused our analysis of food cup entries during second-order cues across the entire 10 s because most behavior (food cup, rear, head jerk) during second-order conditioning tends to be distributed more evenly across the CS period (e.g., Holland, 1977) in contrast to first-order cues, to which most food cup behavior occurs during the last 5 s of the CS (Holland, 1977, 1980).

All rats increased their food cup entries in response to the auditory SOCS+ over the course of three training sessions, while conditioned food cup entries during the pre-SOCS+ period were low and remained relatively stable. In contrast, food cup entries in response to the auditory SOCS− did not increase over the course of three training sessions and remained relatively stable, while food cup entries during the pre-SOCS− periods were also low and remained stable (Figure 5B). Data for SOCS and pre-SOCS responding were analyzed using a within subjects ANOVA, including within subject factor of Session (1–3), SOCS epoch (pre-SOCS, SOCS) and SOCS Discrimination (SOCS−, SOCS+). The analysis of food cup entries showed a main effect SOCS epoch [*F*_(1, 23)_ = 88.4, *p* < 0.05]. There were no main effects of Session or SOCS Discrimination [*F*_(2, 46)_ = 0.3, *p* = 0.8, *F*_(1, 23)_ = 0.2, *p* = 0.7, respectively] but there were significant interactions of Session × SOCS epoch [*F*_(2, 46)_ = 3.2, *p* < 0.05] and Session × SOCS Discrimination [*F*_(2, 46)_=4.3, *p* < 0.05] as well as a significant interaction of Session × SOCS epoch × SOCS Discrimination [*F*_(2, 46)_ = 4.1, *p* < 0.05]. This suggests that we observed acquisition of food cup CR to the auditory SOCS+ associated with the previously reward-paired CS across all rats.

For second-order conditioning we analyzed both compound and elemental FOC trials. The food cup entries during FOC compound and elemental trials are shown in Figures 5C,D, respectively. For both compound and elemental FOC cues, food cup entries during CS+ trials remained high and stable over the course of three training sessions, while food cup entries during the pre-CS+ period were low and remained relatively stable. In contrast, food cup entries in response to the FOC CS− trials over the course of the three training sessions were lower than during the CS+ and remained relatively stable, and food cup entries during the pre-CS− period were also low and remained stable. We analyzed the first-order compound and elemental data using a single within subjects ANOVA, including within subjects factors of Session (1–3), CS epoch (pre-CS, CS), CS Discrimination (CS−, CS+) and Stimulus type (elemental, compound). The analysis of food cup responding showed main effects of Session [*F*_(2, 46)_ = 5.5, *p* < 0.05], CS epoch [*F*_(1, 23)_ = 46.8, *p* < 0.05] and CS Discrimination [*F*_(1, 23)_ = 51.2, *p* < 0.05]. There were significant interactions of Session × CS epoch [*F*_(2, 46)_ = 3.7, *p* < 0.05] and CS epoch × CS Discrimination [*F*_(1, 23)_ = 74.7, *p* < 0.05]. There were no other significant interactions of Session × CS Discrimination [*F*_(2, 46)_ = 0.2, *p* > 0.05] or Session × CS epoch × CS Discrimination [*F*_(2, 46)_ = 0.6, *p* > 0.05]. There were no main effect of Stimulus type [*F*_(1, 23)_ = 0.1, *p* > 0.05] nor any other significant interactions (Stimulus type × Session [*F*_(2, 46)_ = 1.0, *p* > 0.05], Stimulus type × CS epoch [*F*_(1, 23)_ = 0.1, *p* > 0.05], Stimulus type × CS Discrimination [*F*_(1, 23)_ = 0.1, *p* > 0.05], Stimulus type × Session × CS epoch [*F*_(2, 46)_ = 2.4, *p* > 0.05], Stimulus type × Session × CS Discrimination [*F*_(1, 23)_ = 0.4, *p* > 0.05], Stimulus type × CS epoch × CS Discrimination [*F*_(2, 46)_ < 0.1, *p* > 0.05], or Stimulus type × Session × CS epoch × CS Discrimination [*F*_(2, 46)_ = 1.3, *p* > 0.05]). This indicated that food cup responding to light first-order CS+ was maintained even when presented in compound with first-order cues but without food reward. That is, food cup responding to the reward-paired first-order cue did not extinguish during the course of second-order conditioning.

#### Phase III: second-order probe test

**Figure 6:**
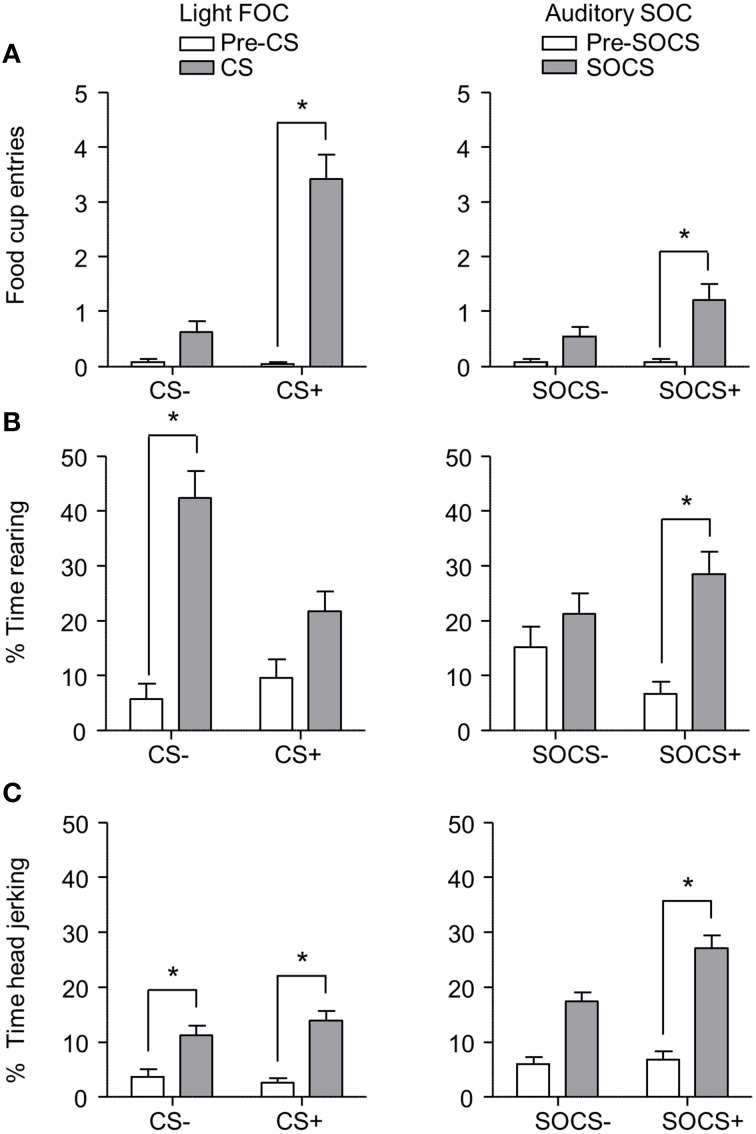
**Exp 2: Phase III: Overall performance during second-order probe test**. **(A)** Number of food cup entries (mean ± SEM) during the last 5 s of the 10 s Pre-CS and CS for the light first-order cue (FOC; CS+, or CS−; left panel) and auditory second-order cues (SOC; SOCS+ or SOCS−; right panel). **(B)** Percent time spent rearing (mean ± SEM) during the first 5 s of the 10 s Pre-CS and CS for the light first-order cues (FOC; CS+ or CS−; left panel) and the total 10 s for the auditory second-order cues (SOC; SOCS+ or SOCS−; right panel). **(C)** Percent time spent head jerking (mean ± SEM) during the total 10 s Pre-CS and CS for the light first-order cues (FOC; CS+ or CS−; left panel) and the total 10 s for the auditory second-order cues (SOC; SOCS+ or SOCS−; right panel). ^*^Different in responding between pre-CS and CS, *p* < 0.05.

After second-order discrimination training, we assessed food cup entries, rearing and head jerk responses to the first-order conditioned stimuli (FOCS) and second-order conditioned stimuli (SOCS) under extinction conditions (Figure 6). We focused our analysis of food cup entries to the last 5 s of the light FOCS, a time when most food cup behavior occurs (Holland, 1977). We focused our analysis of rearing to the first 5 s of a 10 s visual FOCS, as rearing is more frequent during this time period for visual cues (Holland, 1977, 1980). Accordingly, we report food cup entries for only the last 5 s of both FOCS and SOCS trials, and rearing data for only the first 5 s of the FOCS trials. For auditory second-order conditioning, rearing and head jerk were analyzed across the entire 10 s of the SOCS trials because orienting behavior during second-order conditioning tends to be distributed more evenly across the CS periods (Holland, 1977). In accordance with Experiment 1, we presented only results from the first half of the session, the first two trials of each type of stimulus. Data for food cup entries, rearing, and head jerk during first-order and second-order conditioning data were analyzed in six separate within subjects ANOVAs including within subjects factors of CS epoch (pre-CS, CS) and CS Discrimination (CS+, CS−).

Overall we observed more food cup entries to FOCS+ trials relative to FOCS− trials while pre-CS responding during both FOCS+ and FOCS− trials was low (Figure 6A left), and the same pattern of responding was seen on SOCS+ and SOCS− trials (Figure 6A right). The separate analyses of food cup entries for FOCS and SOCS trials showed main effects of CS epoch [*F*_(1, 23)_ = 98.4 and *F*_(1, 23)_ = 16.5, FOC and SOC respectively, *p* < 0.05] and CS Discrimination [*F*_(1, 23)_ = 96.0 and *F*_(1, 23)_ = 5.9, FOC and SOC respectively, *p* < 0.05], as well as a CS epoch × CS Discrimination interaction [*F*_(1, 23)_ = 88.7, *p* < 0.05].

Inconsistent with rearing being primarily considered a conditioned response to light cues, percent time spent rearing during light stimuli throughout probe test was lower to FOCS+ relative to FOCS− trials. Pre-CS responding during both FOCS+ and FOCS− trials was also low (Figure 6B left). In contrast, percent time spent rearing was greater to auditory SOCS+ trials relative to SOCS− trials while pre-CS responding during both SOCS+ and SOCS− trials was low (Figure 6B right). The analysis for percent time spent rearing during light FOCS and the during auditory SOCS trials showed main effects of CS [*F*_(1, 23)_ = 61.1 and *F*_(1, 23)_ = 15.5, FOC and SOC respectively, *p* < 0.05] and CS Discrimination for FOCS trials [*F*_(1, 23)_ = 7.6 for FOC *p* < 0.05]. While there was no main effect of CS Discrimination for SOCS trials [*F*_(1, 23)_ = 0.03, *p* > 0.05], there were significant interactions of CS epoch × CS Discrimination for both FOCS and SOCS trials [*F*_(1, 23)_ = 12.6, and *F*_(1, 23)_ = 6.8, FOCS and SOCS respectively, *p* < 0.05].

Consistent with head jerk being primarily considered a conditioned response to auditory cues, the percent time spent head jerking was similar for light FOCS+ and FOCS− trials relative to pre-CS responding for both FOCS+ and FOCS− (Figure 6C left), and more time was spent head jerking to auditory SOCS+ trials relative to SOCS− trials. Percent time spent head jerking during pre-CS for both SOCS+ and SOCS− cues was low (Figure 6C right). While the analysis for percent time spent head jerking during both FOCS and SOCS trials, showed main effects of CS epoch [*F*_(1, 23)_ = 41.6 and *F*_(1, 23)_ = 83.4, FOC and SOC respectively, *p* < 0.05] there was only a main effect of CS Discrimination for SOCS trials [*F*_(1, 23)_ = 7.6 *p* < 0.05]. The CS epoch × CS Discrimination interaction only reached significance for SOCS trials [*F*_(1, 23)_ = 6.6, *p* < 0.05].

#### Phase IV: lever autoshaping procedure (sign-tracking screening procedure)

**Figure 7:**
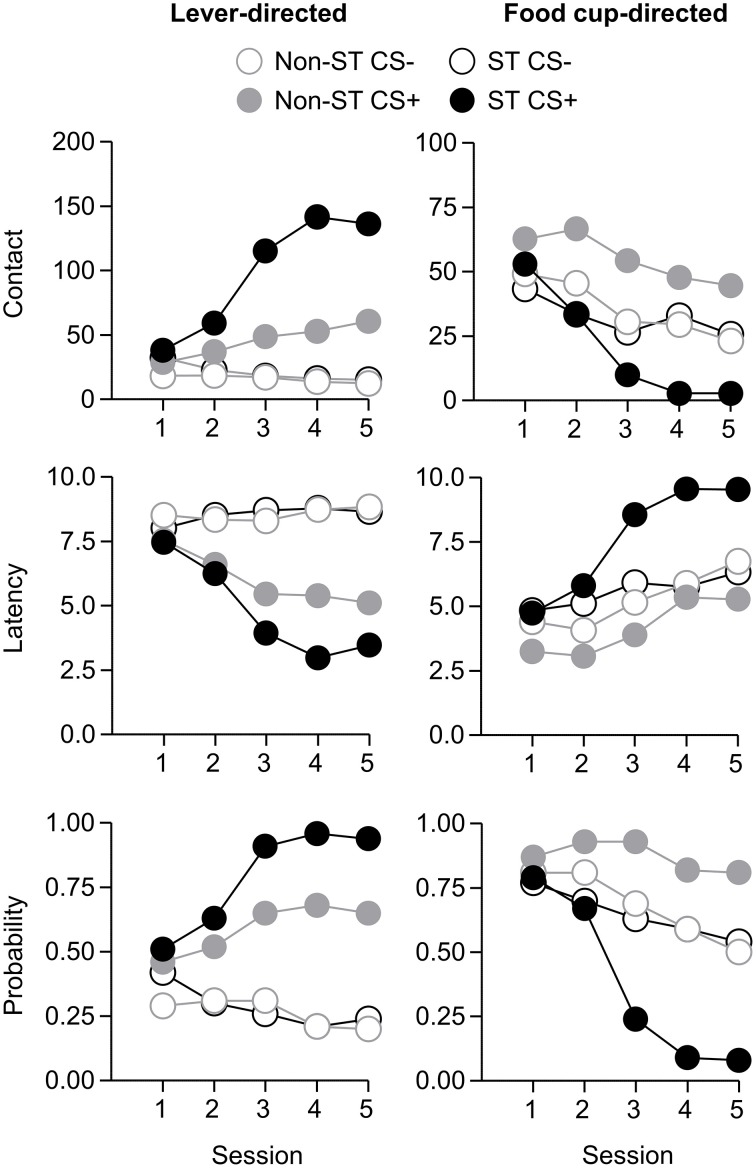
**Experiment 2: Phase IV: Performance during lever autoshaping procedure (Sign-tracking screening procedure)**. Data are mean ± SEM on three different lever-directed (left) and food cup-directed (right) measures. Number of lever and food cup contacts (top row), latency to contact lever or food cup (middle row) and probability of contacting lever or food cup (bottom row).

After the probe test, we screened rats in the lever autoshaping procedure to determine their Tracking tendency. Rats' performance on three lever measures (contact, latency, and probability) and three food cup measures (contact, latency, and probability) is shown in Figure 7. Due to a food cup malfunction during the first session of lever autoshaping that resulted in food cup responding that was greater than three standard deviations outside of the mean for four rats, food cup data from those four rats for all food cup measures were excluded. To maintain the most accurate graphical representation of the lever- and food cup-directed behaviors across all rats we only eliminated this food cup data during the first lever autoshaping session in which there was a malfunction, shown in Figure 7. To maintain integrity of our within subject statistical analysis the data for food cup measures of those four rats was excluded across all five autoshaping sessions. We analyzed the data using six separate sets of mixed repeated measures ANOVAs, using within subject factors of CS (CS−, CS+) and Session (1-5) and between subjects factor of Tracking group (non-ST, ST). The six analyses were on three lever measures (contact, latency, and probability) and three food cup measures (contact, latency, and probability). The main effects and interactions are reported in Table 3. Importantly, as in Experiment 1, the critical CS × Session × Tracking group interactions were significant for all six measures of CR.

**Table 3:** **Experiment 2. Phase IV: Lever autoshaping procedure, summary table of analyses for lever and food cup measures (contact, latency, and probability)**.

**Effect**	**Degrees of freedom**	**Lever**						**Degrees of freedom**	**Food cup**					
		**Contact**		**Latency**		**Probability**			**Contact**		**Latency**		**Probability**	
		***F***	***p***	***F***	***p***	***F***	***p***		***F***	***p***	***F***	***p***	***F***	***p***
CS	(1,22)	52.57	< 0.001	69.21	< 0.001	97.46	< 0.001	(1,18)	0.88	0.36	1.86	0.2	0.53	0.48
Session	(4,88)	10.78	< 0.001	14.25	< 0.001	3.71	< 0.01	(4,72)	28.07	< 0.001	39.20	< 0.001	39.52	< 0.001
Tracking group	(1,22)	12.48	< 0.01	1.48	0.24	2.70	0.11	(1,18)	24.41	< 0.001	33.07	< 0.001	98.75	< 0.001
CS × Session	(4,88)	29.25	< 0.001	38.89	< 0.001	29.95	< 0.001	(4,72)	6.19	< 0.001	9.02	< 0.001	3.46	0.01
CS × Session × Tracking	(4,88)	9.41	< 0.001	4.51	< 0.01	4.33	< 0.01	(4,72)	3.59	0.01	4.28	0.01	12.26	< 0.001

### Lack of individual differences in second-order conditioning probe test

**Figure 8:**
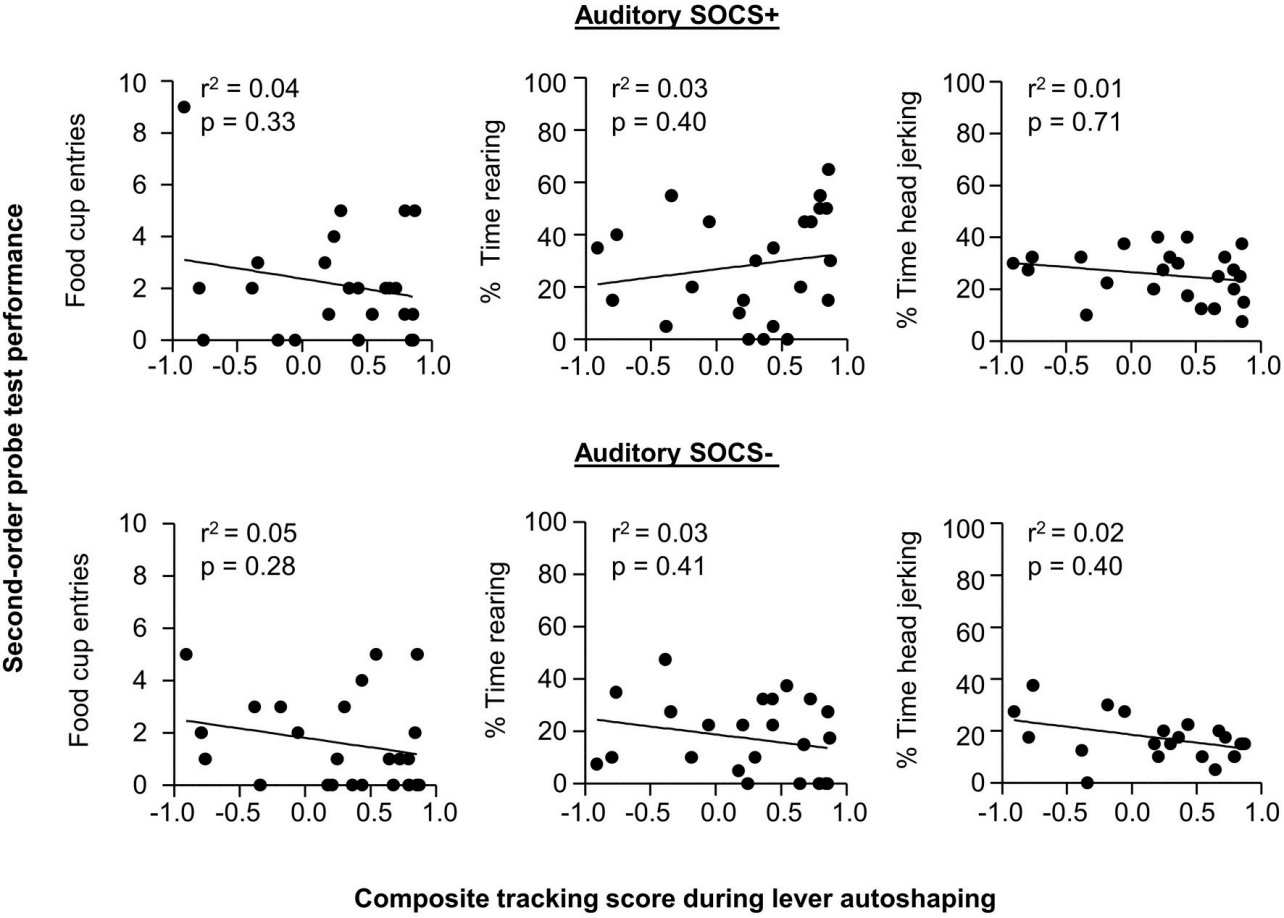
**Experiment 2: Phase IV: Correlation between performance during second-order probe test with tracking tendency during lever autoshaping**. Correlations for auditory SOCS+ (top row) and auditory SOCS− (bottom row) using the measure of food cup entries during second-order cue period (last 5 s of the SOCS) with composite tracking score determined from lever autoshaping (left), using the measure of percent time spent rearing during second-order cue period (total 10 s of the SOCS) with composite tracking score determined from lever autoshaping (middle) and using measure of percent time spent head jerking during second-order cue period (total 10 s of the SOCS) with composite tracking score determined from lever autoshaping (right).

Given our *a priori* prediction that there is individual variability in responding to second-order cues as it relates to tracking tendency, we conducted linear correlation analyses using food cup, rearing, and head jerk CR during the SOCS trials during second-order conditioning probe test and the later determined composite tracking score (as assessed across lever autoshaping, Figure 7). Similar to previous studies (Holland, 1977) we analyzed food cup behavior during the last 5 s of a 10 s CS and we analyzed head jerk and rearing across the entire 10 s of the CS because most orienting behavior during second-order cues tends to be evenly distributed across the CS periods (e.g., Holland, 1977). We did not observe any significant correlations between any of the second-order conditioning measures (food cup, rearing or head jerking) to the SOCS+ (an auditory second-order cue associated with the previously reward-paired first-order cue) and the later determined composite tracking score [SOCS+ and tracking score; *r*^2^ = 0.04, *r*^2^ = 0.05, *r*^2^ = 0.05, food cup, rearing, and head jerk respectively *p* > 0.05, Figure 8 (top row)]. Furthermore, we found no correlation between second-order conditioned responses to the SOCS− (an auditory second-order cue associated with the unrewarded first-order cue) and the later determined composite tracking score (SOCS− and tracking score; *r*^2^ = 0.05, *r*^2^ = 0.06, *r*^2^ = 0.02, food cup, rearing and headjerk, respectively *p* > 0.05, Figure 8 (bottom row). Taken together, these results suggested that there is no relationship between CR to the auditory second-order SOCS+ and tracking tendency during lever autoshaping. To confirm that both sign- and non-sign tracking rats expressed the second-order CS discrimination, we include in the Supplementary Materials parallel analyses to Experiment 1 using between subjects factor of Tracking tendency (see Supplementary Figures S1, S2, S3 and accompanying text). This analysis confirmed the negative finding that variability in Pavlovian incentive learning as assessed by second-order conditioning does not relate to tracking phenotype.

## Discussion

We found in Experiment 1 that performance during outcome devaluation probe test correlated with subsequent tracking tendency, such that sign-tracking rats that formed the food-illness association during the CTA phase failed to reduce responding to the light CS during probe test. We found that the marginally significant overall outcome devaluation effect, that is, the difference between paired and unpaired groups, was carried exclusively by the non-sign-tracking rats, which reduce food cup responding to the light CS after devaluation of the food. This phenotypic difference was not due to differences between tracking groups in acquisition of the light-food association or the food-illness association, as both groups similarly acquired these associations in light conditioning and CTA phases, respectively. Nor was this phenotypic difference the result of a non-specific difference in conditioned food cup responding or extinction of that response during probe test, as evidenced by a failure to see any differences between tracking groups in the unpaired conditions. Therefore, the failure of sign-trackers to suppress CR after reward devaluation is likely due to an inability to use stimulus-outcome associations to guide appropriate responding to CS based on the current value of the US. Notably the finding from our study, which assesses sensitivity to outcome devaluation prior to and outside of the context of autoshaping, stands in contrast to prior studies, which find that several lever directed behaviors are sensitive to outcome devaluation when the US devaluation occurs in or just after exposure to the autoshaping context itself (Cleland and Davey, 1982; Derman and Delamater, 2014). We suggest that the inflexibility observed in the present study is an additional feature of the sign-tracking phenotype, which is consistent with a recent study that observes resistance to Pavlovian extinction in sign-trackers (Ahrens et al., 2015). With relevance to inflexibility in drug-seeking, a prior study demonstrates that sign-trackers respond more than goal-trackers to cocaine-paired cues even in the presence of an aversive shock barrier (Saunders and Robinson, 2013). Here we demonstrated that even prior to drug experience, the later determined sign-tracking rats showed difficulty adjusting their cue-driven natural reward seeking behavior after the reward had been devalued. However, a failure to display a flexible reduction in CR after outcome devaluation could be explained instead by enhanced incentive value attributed to the initially appetitive cue, for which enhanced stimulus-response associations would effectively mask any stimulus-outcome driven learning after outcome devaluation.

To address this possibility, in Experiment 2, we examined whether individual variability in the expression of learned appetitive incentive value, as assessed by Pavlovian second-order conditioning, predicts the tracking phenotype. We observed evidence for second-order conditioning across all rats with three different measures of CR (food cup, rearing, head jerk). Performance during second-order conditioning probe test did not correlate with tracking tendency. Despite the lack of a relationship between these factors, we confirmed that both tracking groups expressed second-order cue discrimination in food cup and head jerk CR. Taken together, results from Experiment 1 and 2 suggest that sign- and non-sign-tracking rats learn equally well to attribute value to the previously rewarded first-order CS, which is then similarly able to support second-order conditioning to a novel auditory cue. Thus, sign-tracking rats appear to have specific difficulty displaying flexible behavior to a first-order CS when the US associated with it is devalued.

### Theoretical and methodological considerations

Here we used Pavlovian outcome devaluation and second-order conditioning procedures to test two forms of incentive learning. In outcome devaluation the acquired appetitive incentive value of the US was manipulated through pairing with an aversive experience. Prior work suggests that the reduction in CR after outcome devaluation are dependent on stimulus-outcome (S-O) associations mediated by a CS−evoked representation of current value of the US (Holland and Straub, 1979; Colwill and Motzkin, 1994; Gallagher et al., 1999; Pickens et al., 2003). In second-order conditioning the acquired appetitive incentive value of the first-order CS was tested directly by pairing with a novel second-order CS. Previous demonstrations of second-order conditioning assert that a change in CR to a second-order cue is either the result of that cue forming a stimulus-stimulus (S-S) association with a first-order cue (Rizley and Rescorla, 1972; Holland and Straub, 1979) or is dependent on stimulus-response (S-R) associations evoked by the second-order cue that are independent of the first-order cue. The latter S-R associative mechanism is evidence for Pavlovian incentive learning that is insensitive to extinction of the first-order cue or to reward devaluation (Holland and Rescorla, 1975; Holland and Straub, 1979; Holland, 1981; McDannald et al., 2013, but also see Rizley and Rescorla, 1972; Rescorla, 1973; Rashotte et al., 1977; Rescorla, 1980; Nairne and Rescorla, 1981; Rescorla, 1982).

While we did not assess which associative mechanism supports learning in our second-order conditioning procedure, the different types of conditioned response (e.g., food cup entry, head jerk, rearing) can inform whether second-order responding is mediated by S-R or S-S associations (Holland and Rescorla, 1975; Holland and Straub, 1979; McDannald et al., 2013). For instance, acquisition food cup entry and head jerk responses to second-order cues are evidence for S-R associations, while acquisition of rearing is evidence for S-S associations. We found increased food cup, head jerk, and rearing to the second-order SOCS+ at test, suggesting the formation of both S-R and S-S associations. However, because we did not see evidence for successful discrimination of first-order cues with rearing we cannot confirm successful S-S driven responding to second-order cues. In addition, if second-order conditioning in our study relied on S-S associations, which are more dependent on the current incentive value of the first-order stimulus than are S-R associations, we would have expected to see a similar relationship between second-order conditioning probe test and tracking tendency to what we observed in the outcome devaluation experiment. Thus, we infer that S-R associations, which are evidence for incentive learning that is insensitive to reward devaluation, are likely the key mechanism mediating learning in our second-order conditioning procedure (Holland and Rescorla, 1975; McDannald et al., 2013).

Another consideration is that we did not observe the tracking-related differences in incentive learning previously identified with conditioned reinforcement procedures (Robinson and Flagel, 2009; Yager and Robinson, 2010, 2013). Previous studies have found that sign-tracking rats show greater conditioned reinforcement effects than goal-tracking rats (Robinson and Flagel, 2009; Yager and Robinson, 2013; Yager et al., 2015), that is, for sign-tracking but not goal-tracking rats, a previously reward-associated Pavlovian cue alone serves as a better reinforcer for the acquisition of a new conditioned instrumental response. It has been suggested from this, together with the observation that Pavlovian lever cues attract sign-trackers to a greater degree than goal-trackers, that sign-trackers attribute greater incentive salience to reward-predictive cues (Robinson and Flagel, 2009). This conclusion stands in contrast to the present results in which we found sign- and non-sign-trackers learn equally well to attribute incentive value to the previously rewarded first-order CS, which is then able to support conditioning to a second-order cue.

There are several theoretical and methodological considerations that may account for this disparity. The first is that two different procedures, conditioned reinforcement and second-order conditioning, have been used to examine the ability of previously reward-paired cue to support acquisition of CR in new associative learning. Importantly, the associative mechanisms that support CR and the form of the conditioned response itself differ in these two procedures. In conditioned reinforcement, instrumental action results in the reward-predictive stimulus, whereas in second-order conditioning a Pavlovian second-order stimulus precedes the reward-predictive stimulus, independent of action. Individual rats may differ in the extent to which a Pavlovian cue can support Pavlovian vs. instrumental incentive learning known to be mediated by different associative learning mechanisms (Lopez et al., 1992; Corbit and Balleine, 2003).

Another methodological difference between this and prior sign-tracking studies examining incentive learning, is that we observed fewer goal-tracking rats, and thus categorize behavior either as sign- or non-sign-tracking. Prior studies using similar two-lever autoshaping procedures (CS+, CS−) to the one used in the present study have also proven very effective in generating sign-tracking in rats (Boakes, 1977; Davey and Cleland, 1982; Kearns and Weiss, 2004; Holland et al., 2014). Prior studies employing single-lever autoshaping typically observe more bimodal distributions of tracking behavior and thus focus the comparison of individual differences in conditioned reinforcement to the two extremes of the tracking continuum, sign- and goal-tracking (Robinson and Flagel, 2009; Saunders and Robinson, 2010; Yager and Robinson, 2010, 2013; Yager et al., 2015). While we observed that the behavior of the non-sign-tracking group is significantly different than the behavior of sign-tracking group (Figures 2, 7; Tables 2, 3), the pattern of behavior in non-sign-trackers during lever autoshaping closely resembles that of intermediate rats (Flagel et al., 2009), and thus it is possible that our use of non-sign-trackers prevents us from observing differences in expression of previously acquired Pavlovian incentive value that relate to tracking phenotype. However, the sign- and non-sign-tracking behavioral distinction used here was sufficient to observe differences between the two tracking groups in learning when incentive value changes as assessed by outcome devaluation.

Finally, in this study we determined the individual rats' tracking phenotype after assessing two forms of Pavlovian incentive learning, in contrast to prior studies in which sign- and goal-trackers are identified prior to incentive learning behavioral assessments (Flagel et al., 2009; Robinson and Flagel, 2009; Saunders and Robinson, 2010; Yager and Robinson, 2010, 2013; Yager et al., 2015). Here the goal was to limit differences in the individual rats' experience with conditioned and unconditioned stimuli in order to identify whether individual differences in incentive learning mapped onto the tracking phenotypes. We did not see evidence for differences between tracking groups in food cup or rearing (data not shown) behaviors during FOC of the light-food association. Because our assessment of incentive learning occurred in rats that have very similar experience with conditioned and unconditioned stimuli we may procedurally limit our ability to observe phenotypic differences driven by first-order incentive cues to support new associative learning. However, in so much as tracking phenotype is a behavioral trait and not a behavioral state, the time at which tracking phenotype is identified would not be expected to drive differences between results of our study and others.

### Candidate brain mechanisms underlying individual differences in incentive learning

The behavioral results of the present study suggest that both sign- and non-sign-tracking rats attribute similar levels of appetitive incentive value to reward-paired cues, while only non-sign-tracking rats are able to flexibly adjust behavior in response to reward-paired cues for which the associated reward had been devalued. The brain circuits mediating Pavlovian outcome devaluation, second-order conditioning, and sign-tracking have considerable overlap. The functional impact of lesioning or disrupting activity in basolateral amygdala (BLA) and nucleus accumbens (NAc) has been demonstrated in each of the paradigms used in the present study. Just as pre-training NAc lesions impair single-reinforcer outcome devaluation (Singh et al., 2010) and acquisition of second-order conditioning (McDannald et al., 2013), they also impair acquisition but not maintenance of sign-tracking behavior (Chang et al., 2012), which is consistent for a role for NAc in acquisition of initial incentive value [however see Chang and Holland (2013) for lack of core and shell alone effects in lever-directed behavior]. Pre-training BLA lesions impair acquisition of incentive value to first-order cues in both outcome devaluation and second-order conditioning procedures (Hatfield et al., 1996). In contrast BLA lesions do not interfere with the acquisition of sign-tracking behavior, but instead impact the maintenance of previously acquired sign-tracking behavior observed during lever autoshaping (Chang et al., 2012). Disconnection lesions of BLA and NAc that eliminate communication between these two areas impair both second-order conditioning (Setlow et al., 2002) and acquisition and maintenance of sign-tracking behavior (Chang et al., 2012), which shows a common function for BLA to NAc circuit for mediating attribution of incentive value to conditioned stimuli.

While caution should be taken when attempting to infer from our behavioral results what brain regions might account for individual differences reported here, the present finding of intact reward devaluation effects in non-sign-trackers, but not in sign-trackers, suggests that BLA's reciprocal interactions with more specialized areas, such as orbitofrontal cortex or insular cortex, known to be critical for the expression of stimulus-outcome learning and goal-directed action (Pickens et al., 2003; Johnson et al., 2009; Parkes and Balleine, 2013) may be differentially involved in the two tracking groups. The interaction between insular cortex and NAc in retrieval of incentive value for goal-directed action has also been established (Parkes et al., 2015) and may be of interest with relevance to individual differences.

### Relevance of tracking-related individual differences for understanding addiction vulnerability

Rodent studies that evaluate individual differences in sign- and goal-tracking behavior have demonstrated that heightened incentive motivation for natural rewards serves as an informative predictor of heightened motivation for drug rewards (Tomie, 1996; Flagel et al., 2009; Robinson and Flagel, 2009; Saunders and Robinson, 2010; Saunders et al., 2013; Yager and Robinson, 2013; Yager et al., 2015). Such pre-clinical procedures aimed at assessing behavioral markers of addiction-vulnerable individuals prior to drug-exposure may have relevance for human addiction. A promising recent study establishes a paradigm for assessing sign- and goal-tracking behaviors in humans (Garofalo and di Pellegrino, 2015), however the link between this procedure and human addiction has yet to be established.

A prominent theme in the addiction field is to understand whether the aberrant behavior of the addicted individual existed prior to drug-experience or whether it was drug-induced. The behavioral results presented here showed that inflexibility to changes in incentive value are evident prior to drug-experience in sign-tracking individuals, for which previous studies have shown have a greater sensitivity to drug-associated discrete cues. Studies directly examining the effects of amphetamine exposure on sign- and goal-tracking behaviors are mixed, sometimes resulting in more sign-tracking behaviors (Doremus-Fitzwater and Spear, 2011; Robinson et al., 2015) or in other studies more goal-tracking behaviors (Simon et al., 2009; Holden and Peoples, 2010). With relevance to the current study, prior cocaine exposure interferes with both stimulus-outcome mediated behavior in outcome devaluation (Schoenbaum and Setlow, 2005) and acquisition and use of learned incentive value to support second-order conditioning (Saddoris and Carelli, 2014). Taken together, it is likely a complex interplay of pre-existing individual differences that predispose addiction vulnerability together with drug-induced neuroadaptations that drive the seemingly aberrant behavior of drug addicted individuals. Here we used classic conditioning procedures with well-defined psychological and neurobiological underpinnings in order to determine whether individual variability in incentive processes map in a meaningful way onto the tracking phenotypes. Accounting for individual differences is likely a useful tool for understanding the brain basis for variability in natural and drug-reward seeking behaviors.

## Author contributions

YC and HN contributed equally to this work. YC, HN, and KF acquired the data; YC and HN analyzed the data; DC, YC, and HN designed the experiments and interpreted the data; DC conceived and supervised the project; DC, YC, HN, and KF contributed to the write-up of the final version.

## Funding

NIDA IRP.

### Conflict of interest statement

The authors declare that the research was conducted in the absence of any commercial or financial relationships that could be construed as a potential conflict of interest.
