# Supplementary material for: Individual variability in behavioral flexibility predicts sign-tracking tendency
Source: Front Behav Neurosci. 2015 Nov 3;9:289. doi: 10.3389/fnbeh.2015.00289 (PMC4630296; doi:10.3389/fnbeh.2015.00289)
Supplement: Supplementary file 1 [file DataSheet1.DOC]

Supplementary Material

Individual variability in behavioral flexibility predicts sign-tracking tendency

Helen M. Nasser^1,2^, Yu-Wei Chen^1^, Kimberly Fiscella^1^, Donna J. Calu*^1,2^

**Correspondence:** Donna J. Calu: [dcalu@som.umaryland.edu](mailto:dcalu@som.umaryland.edu)

Department of Anatomy & Neurobiology

University of Maryland School of Medicine

20 Penn Street – HSFII Room S263

Baltimore, MD 21201

**Supplementary Figure 1**. *Exp. 2; Phase I: Performance during Pavlovian first-order light discrimination conditioning separated by later determined tracking tendency.* Number of food cup entries (mean±SEM) during the last 5 s of the 10 s CS periods of a rewarded light CS+ (blinking or steady, counterbalanced) predictive of food or a non-reinforced light CS- (blinking or steady, counterbalanced) predictive of no food.

All rats acquired the CS discrimination of the first-order lights cues, as evidenced by no differences sign-tracking vs. non-sign-tracking tendency. We analyzed the data using a mixed ANOVA, with between subject factor of Tracking tendency (non-ST, ST) and using within subjects factors of Session (1-12), CS epoch (Pre-CS, CS), and CS Discrimination (CS-, CS+). There were main effects of Session (F(11,242)=8.3, p<0.05), CS epoch (F(1,22)=335.5, p<0.05), and CS Discrimination (F(1,22)=143.4, p<0.05). There were significant interactions of Session x CS epoch (F(11,242)=22.3, p<0.05), Session x CS Discrimination (F(11,242)=11.8, p<0.05) and CS epoch x CS Discrimination (F(1,22)=225.7, p<0.05) as well as a significant Session x CS epoch x CS Discrimination interaction (F(11,242)=12.0, p<0.05). There were no significant main effect of Tracking tendency (F(1,22)=3.3, p=0.08) or interactions of Tracking tendency (Tracking tendency x Session (F(11,242)=0.4, p>0.05), Tracking tendency x CS epoch (F(1,22)=0.3, p>0.05), Tracking tendency x CS Discrimination (F(1,22)=2.5, p>0.05), Tracking tendency x Session x CS epoch (F(11,242)=0.7, p>0.05), Tracking tendency x Session x Discrimination (F(11,242)=0.4, p>0.05), Tracking tendency x CS epoch x CS Discrimination (F(11,242)=3.5, p=0.08), or Tracking tendency x Session, CS epoch x CS Discrimination (F(11,242)=0.8, p<0.05).


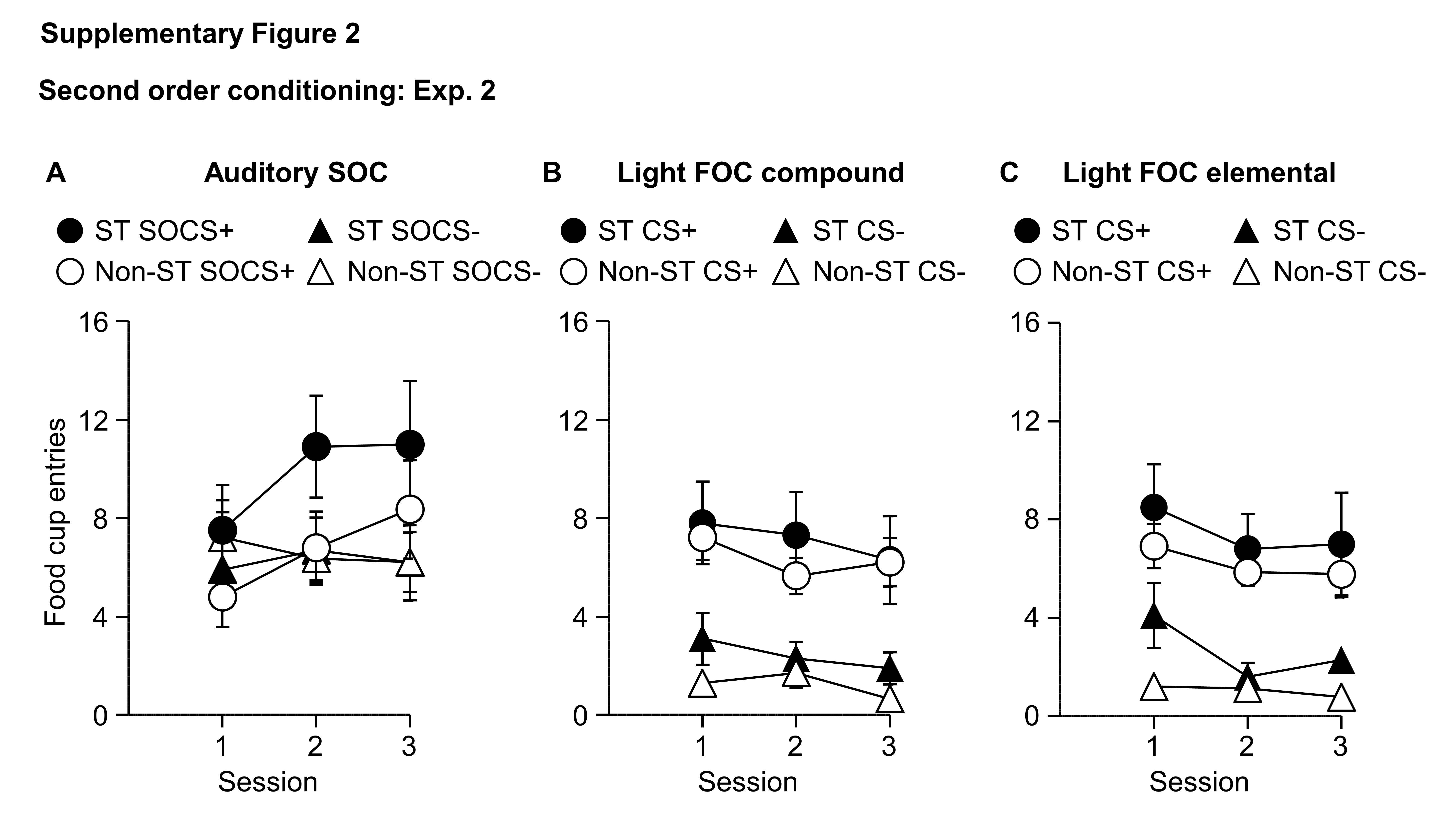
**Supplementary Figure 2.** *Exp. 2; Phase II: Performance during Pavlovian second-order auditory discrimination conditioning separated by later determined tracking tendency.* **(A).** Number of food cup entries (mean±SEM) during the total 10 s of the SOCS periods for the auditory second-order cues (SOCS+ or SOCS-). **(B)** Number of food cup entries (mean±SEM) during the last 5 s of the 10 s CS for the light first-order cues (FOC compound; CS+ or CS-) when it was presented in compound with the SOCS+ or SOCS-. **(C)** Number of food cup entries (mean±SEM) during the last 5 s of the 10 s CS for the light first-order cues (FOC element; CS+ or CS-) element, ‘reminder trials,’ when these light cues were rewarded or unrewarded, respectively.

All rats increased their food cup entries in response to the auditory SOCS+ over the course of three training sessions, while food cup entries in response to the auditory SOCS- over the course of three training sessions were lower than during the SOCS+ and remained relatively stable. Data for SOCS and pre-SOCS responding were analyzed using a mixed ANOVA, using between subjects factor of Tracking tendency (non-ST, ST) and within subject factor of Session (1-3), SOCS epoch (pre-SOCS, SOCS) and SOCS Discrimination (SOCS-, SOCS+). The analysis of food cup responding showed a main effect SOCS epoch (F(1,22)=86.1, p<0.05). There were no main effects of Session or SOCS Discrimination (F(1,22)=2.0, p =0.2, F(2,44)=0.3, p=0.7, F(1,22)=0.3, p=0.6, respectively). There were significant interactions of Session x SOCS epoch (F(2,44)=3.3, p<0.05) and Session x SOCS Discrimination (F(2,44)=3.7, p<0.05) as well as a significant interaction of Session x SOCS epoch x SOCS Discrimination (F(2,44)=4.1, p<0.05). There were no significant main effects of Tracking tendency (F(1,22)=2.0, p =0.2) nor any other interactions (Tracking tendency x Session (F(2,44)=0.1, p>0.05), Tracking tendency x SOCS epoch (F(1,22)=0.6, p>0.05), Tracking tendency x SOCS Discrimination (F(1,22)<0.1, p>0.05), Tracking tendency x Session x SOCS epoch (F(2,44)=1.4, p>0.05), Tracking tendency x Session x SOCS Discrimination (F(2,44)=0.8, p>0.05), Tracking tendency x SOCS epoch x SOCS Discrimination (F(1,22)=2.0, p>0.05), or Tracking tendency x Session x SOCS epoch x SOCS Discrimination (F(2,44)=1.2, p>0.05).

This suggests there were no significant differences in acquisition of second-order discrimination for the two tracking groups. For both elemental and compound FOC cues food cup entries during CS+ trials remained high and stable over the course of three training sessions, and there were no significant differences in Tracking tendency. We analyzed the first-order elemental and compound data with a mixed ANOVA, using between subjects factor of Tracking tendency (non-ST, ST) and within subject factors of Session (1-3), CS epoch (pre-CS, CS), CS Discrimination (CS-, CS+) and Stimulus type (elemental, compound). The analysis of food cup responding showed main effects of Session (F(2,44)=6.6, p<0.05), CS epoch (F(1,22)=47.1, p<0.05) and CS Discrimination (F(1,22)=57.0, p<0.05). There were significant interactions of Session x CS epoch (F(2,44)=3.5, p<0.05) and CS epoch x CS Discrimination (F(1,22)=69.5, p<0.05). There were no significant main effect of Tracking tendency (F(1,22)=2.2, p=0.2) nor any other significant interaction (Tracking tendency x Session (F(2,44)=1.9, p>0.05), Tracking tendency x SOCS epoch (F(1,22)=0.8, p>0.05), Tracking tendency x SOCS Discrimination (F(1,22)=0.4, p>0.05), Tracking tendency x Session x SOCS epoch (F(2,44)<0.1, p>0.05), Tracking tendency x Session x SOCS Discrimination (F(2,44)=0.9, p>0.05), Tracking tendency x SOCS epoch x SOCS Discrimination (F(1,22)<0.1, p>0.05) or, Tracking tendency x Session x SOCS epoch x SOCS Discrimination (F(2,44)=1.6, p>0.05). There was no main effect of Stimulus type (F(1,22)=0.1, p=0.7). There was a significant interaction of Stimulus type x Session x SOCS epoch (F(2,44)=3.7, p<0.05) which reflects the difference in level of pre-cs responding across the three days of training, however there were no other significant interactions (Stimulus type x Session (F(2,44)=0.9, p>0.05), Stimulus type x SOCS epoch (F(1,22)=0.2, p>0.05), Stimulus type x SOCS Discrimination (F(1,22)=0.1, p>0.05), Stimulus type x Session x SOCS Discrimination (F(2,44)<0.1, p>0.05), Stimulus type x SOCS epoch x SOCS Discrimination (F(1,22)=0.4, p>0.05) or, Stimulus type x Session x SOCS epoch x SOCS Discrimination (F(2,44)=1.4, p>0.05). This indicates that food cup responding to light first-order cues were maintained even when presented in compound without reward. That is, food cup responding to the previously rewarded first-order cue did not extinguish during the course of second-order conditioning in either tracking group.


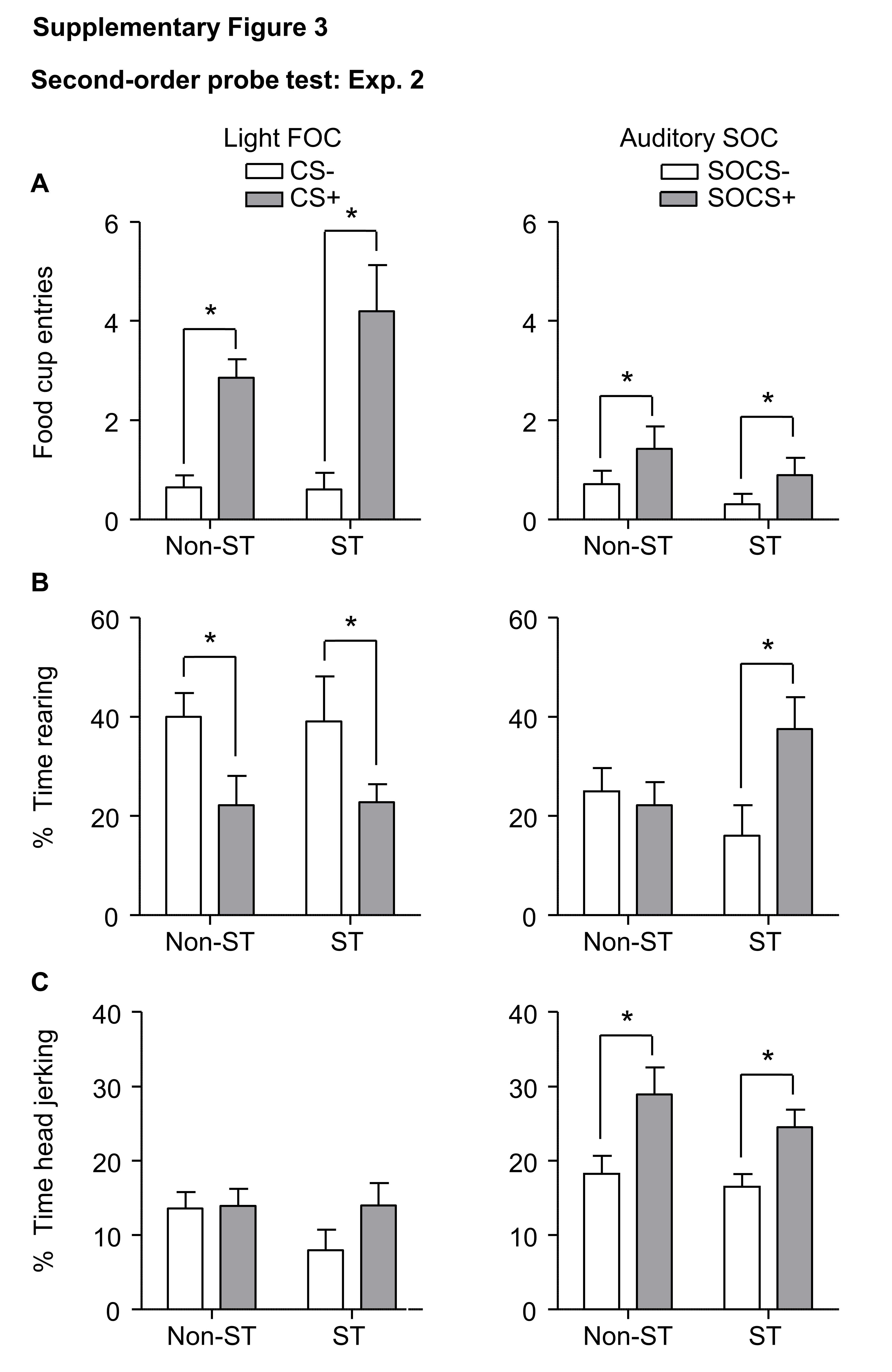


**Supplementary Figure 3**. *Exp 2; Phase III: Performance during probe test separated by later determined tracking tendency.* **(A)** Number of food cup entries (mean±SEM) during the last 5 s of the 10 s CS for the light first-order cue (FOC; CS+ or CS-; left panel) and auditory second-order cues (SOC; SOCS+ or SOCS-; right panel). **(B)** Percent time spent rearing (mean±SEM) during the first 5 s of the 10 s CS for the light first-order cues (FOC; CS+ or CS-; left panel) and the total 10s for the auditory second-order cues (SOC; SOCS+ or SOCS-; right panel). **(C)** Percent time spent head jerking (mean±SEM) during the total 10 s CS for the light first-order cues (FOC; CS+ or CS-; left panel) and the total 10 s for the auditory second-order cues (SOC; SOCS+ or SOCS-; right panel). ^*^ Different in responding between SOCS+ or SOCS- , or CS+ or CS- p<0.05.

We added a between subjects factor of Tracking tendency to the analysis of second-order conditioning probe test performance to first- and second- order cues (FOC and SOC). As explained in the main text, we report food cup entries, rearing and head jerk behavior focused on specific time windows in accordance with Holland, 1977and in an identical manor to that reported in main text analyses. Data for CS and pre-CS responding were analyzed using two separate sets of mixed ANOVAs for FOC and for SOC using between subjects factor or Tracking tendency and within subject factors of CS epoch (pre-CS, CS) and CS Discrimination (CS+, CS-). The analysis of food cup responding showed a significant main effect of CS epoch (F(1,22)=46.5 and F(1,22)=14.7, FOC and SOC respectively, p<0.05) and a significant main effect of CS Discrimination (F(1,22)=58.7 and F(1,22)=5.6, FOC and SOC respectively, p<0.05), as well as, a significant interaction of CS epoch and CS Discrimination for FOC (F(1,22)=53.4, p<0.05) and a close but not significant interaction of CS epoch and CS Discrimination for SOC (F(1,22)=4, p=0.058). In the analysis of food cup entries there were no significant effects of Tracking tendency during either FOC or SOC cues (Fs(1,22)<3.9, ps>0.05).

The analysis for percent time spent rearing to FOC and SOC cues showed a significant main effect of CS epoch (F(1,22)= 56.2 and F(1,22)=14.9, FOC and SOC respectively, p<0.05) and significant main effect of CS Discrimination for FOC cues (F(1,22)=7.4, p<0.05). There was no significant main effect of CS Discrimination for SOC cues (F(1,22)=0.06, p>0.05). There was a significant interaction of CS epoch and CS Discrimination for both FOC and SOC cues (F(1,22)=13, and F(1,22)=7.6, FOC and SOC respectively, p<0.05). There was no significant main effects of Tracking tendency during either FOC or SOC cues (F(1,22)= 0.7, and F(1,22)= 0.3, p>0.05). In the analysis of rearing behavior there was a significant interaction of CS Discrimination and Tracking tendency for SOC cues (F(1,22)= 6.9, p<0.05). There were no other significant interactions with Tracking tendency during either FOC or SOC cues (Fs(1,22)<1.2, ps>0.05).

The analysis for percent time spent head jerking during both FOC and SOC cues, showed a significant main effect of CS epoch (F(1,22)= 38.1 and F(1,22)=81.3, FOC and SOC respectively, p<0.05) and significant main effect of CS Discrimination for SOC cues (F(1,22)=7.1 p<0.05) as well as a significant interaction of CS epoch and CS Discrimination of SOC cues (F(1,22)=5.7, p<0.05). There was no significant main effect of CS Discrimination nor a significant interaction of CS epoch and CS Discrimination for FOC cues in head jerk responding (F(1,22)=0.6 and F(1,22)=1.6, respectively). In the analysis of head jerk behavior there were no significant effects of Tracking tendency during either FOC or SOC cues (Fs(1,22)<2.7, ps>0.05).

We see equal ability for sign-trackers and non-sign-trackers to discriminate between first- and second-order cues across food-cup and head jerking measures. This suggests that both sign-trackers and non-sign-trackers are able learn about the second-order cues. While we see better discrimination in rearing amongst sign-trackers to SOC cues, we also see that sign-trackers and non-sign-trackers both show higher levels of rearing to the first-order CS- relative to the CS+. While we may see a difference in tracking tendency with respect to rearing to second-order cues, the inability to show rearing to the first-order cue previously associated with food (CS+) means that we did not see successful second-order conditioning when looking at this rearing measure.
